# Engineering, Expression, Purification, and Application of Glycosaminoglycan‐Specific Antibodies

**DOI:** 10.1002/cpz1.70358

**Published:** 2026-05-13

**Authors:** Kheerthana Duraivelan, Sriram Sundaravel, Esther N. Njoroge, Robert A. Townley, Ulrich G. Steidl, Hannes E. Bülow, Steven C. Almo, Scott J. Garforth

**Affiliations:** ^1^ Department of Biochemistry Albert Einstein College of Medicine Bronx New York; ^2^ Department of Cell Biology Albert Einstein College of Medicine Bronx New York; ^3^ Department of Oncology Albert Einstein College of Medicine Bronx New York; ^4^ Department of Medicine Albert Einstein College of Medicine Bronx New York; ^5^ Blood Cancer Institute Albert Einstein College of Medicine Bronx New York; ^6^ Montefiore Einstein Comprehensive Cancer Center Albert Einstein College of Medicine Bronx New York; ^7^ Department of Genetics Albert Einstein College of Medicine Bronx New York; ^8^ Dominick P. Purpura Department of Neuroscience Albert Einstein College of Medicine Bronx New York

**Keywords:** glycosaminoglycan, heterologous protein expression and purification, heparan sulfate, single chain variable fragment, scFv

## Abstract

Cluster of differentiation markers have been transformative for defining cell populations and their functional states, but recent work indicates that finer granularity can be achieved by considering the diverse heparan sulfate structures presented on proteoglycans. Heparan sulfates (HSs) are long, unbranched polysaccharides of a repeating disaccharide composed of hexuronic acid and *N*‐acetylglucosamine. HS is attached to core proteins via serine residues. Owing to multiple modifications of the sugar moieties by a set of specific Golgi‐resident modification enzymes, HSs exhibit extraordinary variations in modification patterns. Because these patterns are non‐template based, they display substantial heterogeneity, yet are expressed reproducibly with high spatiotemporal and tissue‐specific selectivity. This complexity of HS modification is, in turn, believed to control extracellular receptor:ligand interactions in a cell‐specific fashion. Conventional methods to study HS structure require specialized expertise and instrumentation, and remain challenging due to the considerable heterogeneity of even tissue‐specific HSs. Here, we describe protocols for the production and implementation of a panel of anti‐HS single‐chain variable fragments (scFvs) based on scFvs originally reported in the 1990s and early 2000s. This panel is an attractive resource for detailed study of HS modification patterns in various physiological processes. Rather than determining HS structure by analytical means, we use this panel to define cells based on the scFvs they bind. We detail practical considerations, strategies, and protocols for the construction, development, expression, purification, and application of the anti‐HS scFv panel from bacterial and mammalian expression systems. © 2026 The Author(s). *Current Protocols* published by Wiley Periodicals LLC.

**Basic Protocol 1**: Expression and purification of anti‐HS scFvs from bacterial expression systems

**Basic protocol 2**: Expression and purification of anti‐HS scFvs from mammalian expression systems

**Basic Protocol 3**: Sortase‐mediated site‐specific labeling of anti‐HS scFvs

**Basic Protocol 4**: *In vitro* HS detection by ELISA

**Basic Protocol 5**: *In vitro* HS detection by flow cytometry using indirect labeling in live Jurkat E6‐1 cells

**Alternate Protocol 1**: *In vitro* HS detection by flow cytometry using indirect labeling in fixed Vero cells

**Alternate Protocol 2**: *In vitro* HS detection by flow cytometry using direct labeling in live Vero cells

**Basic Protocol 6**: *In vitro* HS detection by immunofluorescence in fixed adherent cells

**Alternate Protocol 3**: *In vitro* HS detection by immunofluorescence in suspension cells prior to fixation

## INTRODUCTION

Accurate delineation of cell populations and their functional states is a cornerstone of modern biology, underpinning our understanding of physiology and disease, and informing the development of selective therapies. In this context, cluster of differentiation (CD) markers have been transformative. Recent studies suggest, however, that greater cellular resolution can be achieved by including staining reagents targeting the diverse heparan sulfate (HS) structures presented on proteoglycans. In particular, single‐chain variable fragments (scFvs) against heparan sulfate have been demonstrated to allow stratification of hematopoietic progenitor cells with more granularity than using CD markers alone (Piszczatowski et al., 2022).

**Figure 1:**
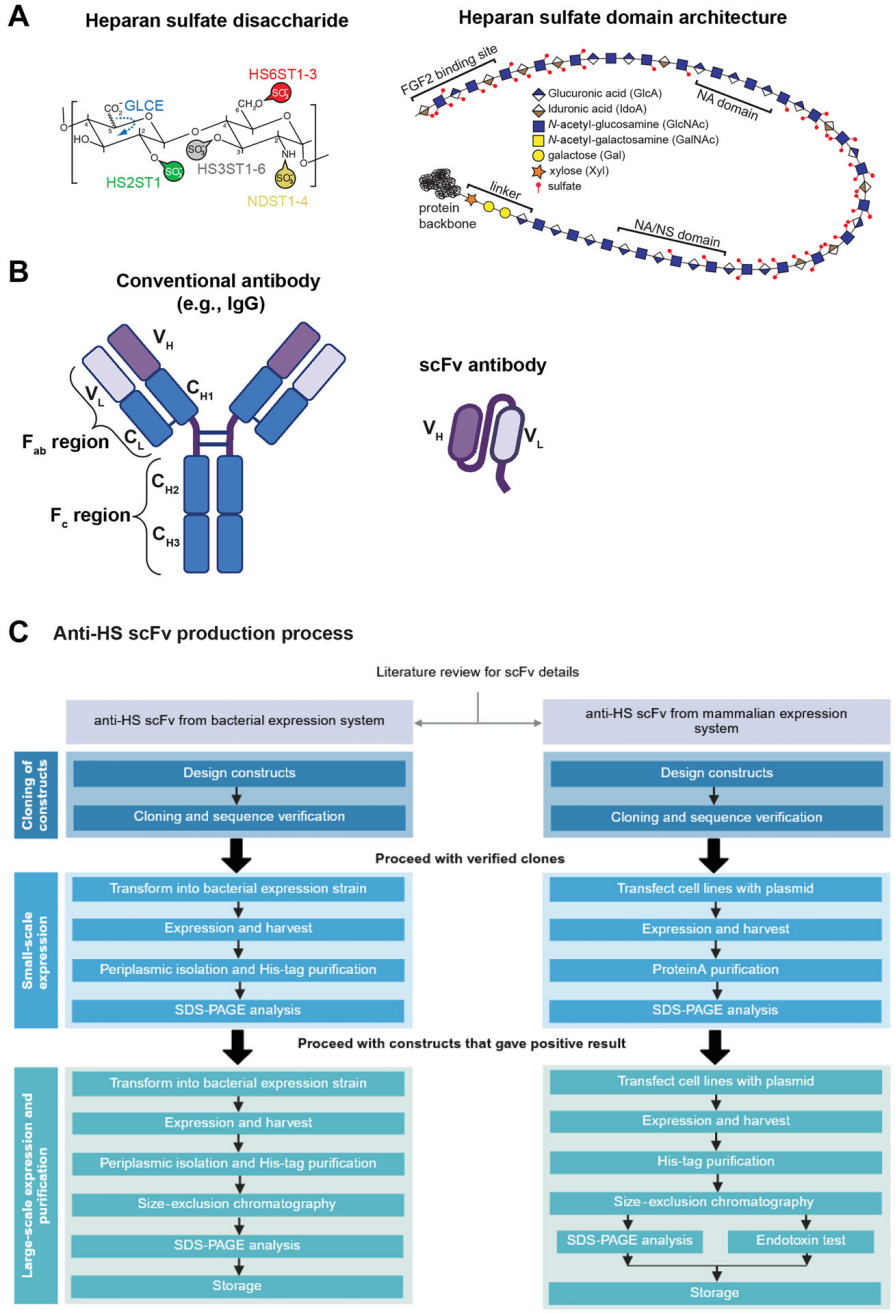
Heparan sulfate proteoglycans and anti‐HS scFv antibodies. (**A**) Schematic representing (left) the basic disaccharide unit that constitutes the HS chain backbone and (right) a single HS chain linked to a core proteoglycan carrying various possible modification patterns. Reprinted from Townley & Bülow (2018) with permission from Elsevier. (**B**) Conventional versus scFv antibody. (**C**) Overview of the scFv production process.

Heparan sulfates are long, unbranched polymers of a disaccharide repeat composed of alternating hexuronic and *N*‐acetylglucosamine residues (Esko & Lindahl, 2001; Esko & Selleck, 2002). Heparan sulfate is covalently attached to core proteins via an invariant tetrasaccharide linker to serine, resulting in HS proteoglycans (Fig. 1A) (Esko & Lindahl, 2001; Esko & Selleck, 2002). During attachment and synthesis of the HS chains in the Golgi apparatus, the glycans undergo a series of modifications, including epimerization of glucuronic to iduronic residues and sulfation of the 2‐OH group of the hexuronic acid as well as the 3‐OH and 6‐OH groups of *N*‐acetylglucosamine (Esko & Lindahl, 2001; Esko & Selleck, 2002). Additionally, *N*‐acetylglucosamine can be deacetylated and sulfated on the amino group. These modifications occur reproducibly but non‐randomly for a given cell type and are not based on a template. Therefore, the resultant HS modification patterns display exceptional molecular diversity (Esko & Lindahl, 2001; Esko & Selleck, 2002). Despite the breadth of the available chemical space, modification patterns are expressed reproducibly with remarkable cellular specificity (Jenniskens et al., 2000; van de Westerlo et al., 2002; van Kuppevelt et al., 1998). In nematodes, some HS modification patterns have been found to be specific to individual cells and conserved across evolution (Attreed et al., 2012, 2016). Heparan sulfates are essential for development and physiology (Bishop et al., 2007; Bülow & Hobert, 2006; Piszczatowski et al., 2024; Sarrazin et al., 2011) and they exert their functions by mediating protein‐protein interactions or modulating protein distribution and localization (Lindahl & Li, 2009; Townley & Bülow, 2018; Xu & Esko, 2014).

Despite these advances, the identification of biologically relevant HS motifs or epitopes and the characterization of HS moieties that are characteristic of a given cell type remain elusive. Great strides have been made in sequencing HS oligosaccharides by using, for instance, mass spectrometric and nanopore sequencing approaches (Liang & Sharp, 2022; Miller et al., 2020; Turnbull et al., 1999; Venkataraman et al., 1999; Wu et al., 2019), but it has proven more challenging to apply these methods to HS from biological sources because of the inherent heterogeneity of the samples owing to the non‐template‐based synthesis in the Golgi. Additionally, these bioanalytical approaches often require specialized instrumentation and expertise. To overcome these challenges, we have implemented a panel of anti‐HS scFvs to define cells by their qualitative and quantitative binding to the anti‐HS scFvs (Piszczatowski et al., 2022, 2024). These anti‐HS scFvs are based on scFvs originally isolated by van Kuppevelt and colleagues (Dennissen et al., 2002; Jenniskens et al., 2000; Smits et al., 2006; van de Westerlo et al., 2002; van Kuppevelt et al., 1998) and are listed in Table 1. Here, we describe protocols for construction, development, expression, and purification of the anti‐HS scFv panel using both bacterial and mammalian expression systems. In addition, we provide proof‐of‐concept protocols to utilize this toolset in high‐throughput approaches, including enzyme‐linked immunosorbent assays, flow cytometry, and immunofluorescence. The data in this article demonstrate that these scFvs can be used in live as well as fixed (e.g., paraformaldehyde‐cross‐linked) cells, showcasing the adaptability of this approach to recognize HS in different experimental workflows.

**Table 1:** Details for Anti‐HS scFv Constructs

Name*^a^*	V_H_ family	V_H_ segment	CDR3	Presumed analog from literature	Originally isolated against*^b^*	References*^c^*
HS001	3	DP‐47	SLRMNGWRAHQ	AO4B08	mskHS	Jenniskens et al. (2000)
HS002	3	DP‐38	GKMKLNR	EV3B2 EW4D2	hlHS/iHep	Dennissen et al. (2002)
HS003	3	DP‐42	GYRPRF	EV3C3	hlHS	Dennissen et al. (2002)
HS004	3	DP‐47	SISMNGVGVRIQ	EV3D1	hlHS	Dennissen et al. (2002)
HS005	3	DP‐38	GRTVGRN	EW3D10 EW3D3	iHep	van de Westerlo et al. (2002)
HS006	3	DP‐38	DRRNTQKTRYRT	EW3E4	iHep	van de Westerlo et al. (2002)
HS007	3	DP‐38	SGRQARQGRFPK	EW3F5	iHep	van de Westerlo et al. (2002)
HS008	3	DP‐38	GGTTRIRK	EW3G6	iHep	van de Westerlo et al. (2002)
HS009	1	DP‐08	GTKLKMTK	EW4A4	iHep	van de Westerlo et al. (2002)
HS010	3	DP‐38	ERNTIRR	EW4A11	iHep	van de Westerlo et al. (2002)
HS011	3	DP‐45	GRLHLPRK	EW4B5	iHep	van de Westerlo et al. (2002)
HS012	1	DP‐08	SSSRHHRLHR	EW4B7	iHep	van de Westerlo et al. (2002)
HS013	1	DP‐25	QRWKPAVTPKLV	EW4B10	iHep	van de Westerlo et al. (2002)
HS014	3	DP‐45	ARMTGHVRNVMI	EW4C10	iHep	van de Westerlo et al. (2002)
HS015	3	DP‐45	PVSHRKWRVTV	EW4D5	iHep	van de Westerlo et al. (2002)
HS016	3	DP‐38	GRRHKLIR	EW4E1	iHep	van de Westerlo et al. (2002)
HS017	3	DP‐38	LRGTKMFRH	EW4E9	iHep	van de Westerlo et al. (2002)
HS018	1	DP‐03	SRKTPKPFMRK	EW4E10	iHep	van de Westerlo et al. (2002)
HS019	3	DP‐42	GARLKR	EW4G1	iHep	van de Westerlo et al. (2002)
HS020	3	DP‐38	GKVKLPN	EW4G2	iHep	van de Westerlo et al. (2002)
HS021	3	DP‐45	GTKKLGK	EW4G10	iHep	van de Westerlo et al. (2002)
HS022	3	DP‐38	GMRPRL	HS3A8 EW3H12 RB4CD12	bkHS/iHep/hsmHS	Jenniskens et al. (2000)
HS023	1	DP‐03	SRKTRKPFMRK	HS3B7	bkHS	Dennissen et al. (2002)
HS024	1	DP‐08	YYHYKVN	HS3G8	bkHS	van Kuppevelt et al. (1998)
HS025	4	DP‐65	WVTEP	HS4A5	bkHS	Dennissen et al. (2002)
HS026	3	DP‐38	GRRLKD	HS4C3	bkHS	van Kuppevelt et al. (1998)
HS027	3	DP‐58	GMRPRL	HS4D4	bkHS	Dennissen et al. (2002)
HS028	3	DP‐42	SLRMNGCGAHQ	HS4D10	bkHS	van Kuppevelt et al. (1998)
HS029	3	DP‐38	HAPLRNTRTNT	HS4E4 RB4CB9	bkHS/hsmHS	Jenniskens et al. (2000)
HS030	3	DP‐38	GSRSSR	LKIV69	bkHS	Wijnhoven et al. (2008)
HS031	3	DP‐47	QKKRPRF	MW3G3	AS	ten Dam et al. (2004)
HS032	3	DP‐53	SGRKGRMR	NS4F5	hlHS	Smits et al. (2010)
HS033	3	DP‐32	RRYALDY	RB4EA12	hsmHS	Jenniskens et al. (2000)
HS034	3	DP‐38	WRNDRQ	MPB49	No known HS epitope, negative control	Smits et al. (2006)
HS035	3	DP‐7	LKQQGIS	AO4B05	mskHS	Jenniskens et al. (2000)
HS036	1	DP‐4	AMTQKKPRKLSL	AO4F12	mskHS	Jenniskens et al. (2000)
HS037	1	DP‐38	SGRKYFRARDDMN	RB4EG12	hsmHS	Jenniskens et al. (2000)

^*a*^ The numbered scFv antibodies (HS001‐HS037) are similar or identical to previously described (legacy) scFvs as indicated in the presumed analog column (see indicated references). scFv antibodies were isolated by panning against different HS preparations as indicated. For the sake of operational simplicity, we numbered the scFvs numerically with HS indicating that they recognize heparan sulfate. Note that some of the reagents described here behave differently than their presumed analogs. For example, we have never observed binding of HS027 to any cells or to bkHS. Therefore, general caution should be exercised when relating experiments obtained using the reagents described here with experiments obtained using legacy reagents. The precise sequences of all scFvs described here will be published elsewhere.

^*b*^ HS preparations against which the presumed analogs were panned: AS, acharan sulfate; bkHS, bovine kidney HS; hlHS, human lung HS; hsmHS, human skeletal muscle HS; iHep, immobilized heparin; mskHS, mouse skeletal muscle HS.

^*c*^ First article to report the specific anti‐HS scFv.

## STRATEGIC PLANNING

### Intended Experimental Uses, Limitations, and Context

The protocols described here are intended to enable researchers to produce the anti‐HS scFv panel and utilize them in studying HS biology in a variety of biological contexts, from cell lines to primary cells. The panel is intended to be used as a complementary tool to existing HS analysis techniques such as mass spectrometry and disaccharide analysis, primarily for mapping and defining cells based on the presence of HS epitopes, the sum of which we refer to as the glycotype.

Reproducibility between different laboratories is an important attribute that may be affected by a number of factors including, but not limited to, instrumentation, sensitivity of detection (e.g., using polyclonal versus monoclonal secondary antibodies or using fluorophores with different quantum yields), and differences between cell‐lines tested, either because of genetic drift or differences in culture conditions. Hence, in the Commentary, we have included heat maps showing the expected fluorescence‐activated cell sorting (FACS) staining patterns for two commonly used cell lines (adherent HEK293 cells and Jurkat E6‐1 cells in suspension), which can be used to benchmark the anti‐HS scFv panel.

### Anti‐HS scFvs

**Figure 2:**
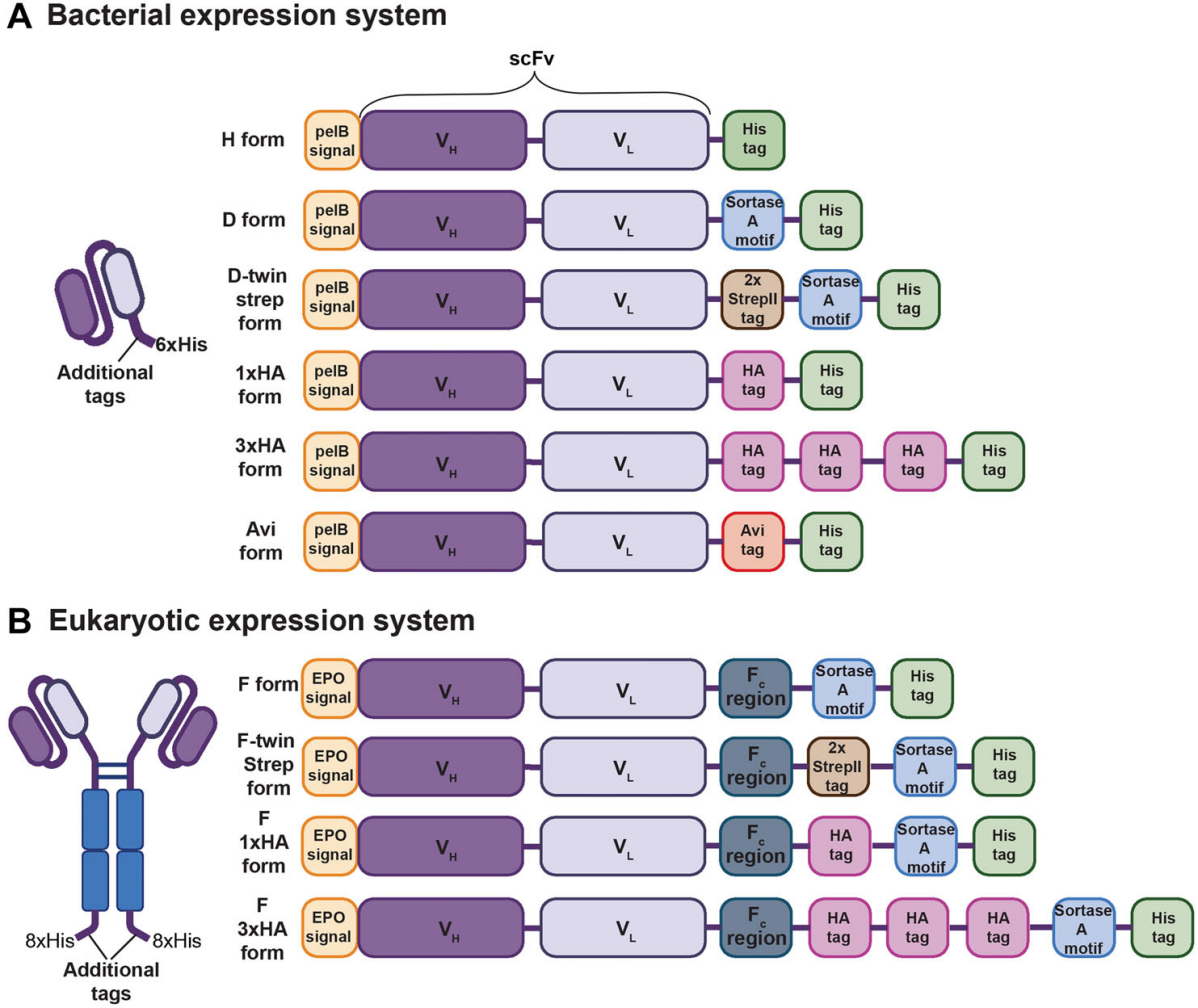
Domain organization of anti‐HS scFv constructs. Variants are expressed in an *E. coli* expression system (**A**) or ExpiCHO mammalian expression system (**B**).

scFv antibodies are synthetic constructs consisting of the variable region of a conventional antibody and are constructed by linking the variable heavy and variable light domains that constitute the epitope‐binding region (Fig. 1B). The anti‐HS scFv antibodies were originally described by van Kuppevelt and colleagues and generated via phage display using heparan sulfates and heparin as the immunogens (Table 1) (Dennissen et al., 2002; Jenniskens et al., 2000; Smits et al., 2006; van de Westerlo et al., 2002; van Kuppevelt et al., 1998). We reconstructed the sequences of these scFvs on the basis of general scFv sequences of the Nissim cDNA library (Marks et al., 1991; Nissim et al., 1994) and the CDR3 sequences reported by van Kuppevelt and colleagues (Dennissen et al., 2002; Jenniskens et al., 2000; Smits et al., 2006; van de Westerlo et al., 2002; van Kuppevelt et al., 1998). The precise sequences of the scFv antibodies described here will be published elsewhere. It should be noted that while the sequences of the anti‐HS scFvs were reconstructed with the utmost care, we cannot guarantee that the sequences are identical to the anti‐HS scFv antibodies originally isolated by van Kuppevelt. Because of this uncertainty, and for the sake of operational simplicity, we have named the anti‐HS scFvs numerically and listed them together with the names of their presumed analogs as indicated in Table 1. Caution must be exercised when relating results obtained with the reagents described here to legacy reagents, and direct experimental comparisons may be necessary. The scFv backbones were then further modified with different tags and functionalizations as described below. A brief overview of the anti‐HS scFv production process is illustrated in Figure 1C, and the domain organizations of the various anti‐HS scFv constructs are depicted in Figure 2.

### Bacterial expression system

*Escherichia coli* expression systems are extensively used for heterologous expression of recombinant proteins as they offer easy genetic manipulation, an array of widely available vectors and media, and relatively low costs (Francis & Page, 2010). Our anti‐HS scFv‐H variant constructs are designed to be expressed in *E. coli* with an N‐terminal pelB signal sequence (MKYLLPTAAAGLLLLAAQPAMA) to direct the protein to the periplasm, followed by the V_H_ and V_L_ segments of the scFv and a C‐terminal 6×His tag (Fig. 2A). Periplasmic expression can improve recombinant protein solubility, increase correct disulfide bond formation, and, due to reduced protease and overall protein content relative to the cytoplasm, improve and simplify protein purification. The expressed protein can then be extracted by selectively disrupting the outer membrane (Ghamghami et al., 2020; Quan et al., 2013). We also designed constructs with alternate tags for additional detection or site‐specific conjugation options (Fig. 2A).

### Mammalian expression system

Our anti‐HS scFv‐F variants are scFvs fused with the Fc region of human IgG1. In addition to providing an increase in valency, the Fc region can impart additional stability to the scFvs (Czajkowsky et al., 2012). Expression from a eukaryotic system also simplifies endotoxin‐free production, enabling the direct use of these scFv‐F variants in pyrogen‐sensitive cell culture or *in vivo* experiments. We utilize the ExpiCHO expression system for production of anti‐HS scFv‐F variants.

The pcDNA3.3 vector, a mammalian vector with target expression driven by the CMV promoter, was initially modified by addition of the β‐2‐microglobulin signal sequence (MSRSVALAVLALLSLSGLEA) to direct protein secretion, and a C‐terminal human IgG1 Fc region followed by a sortase A motif, 8×His tag, and stop codon. We then cloned the anti‐HS scFv segments between the signal peptide and the Fc region (Fig. 2B).

The 8×His tag and Fc region serve as both purification and detection tags, while the sortase A motif can be used for sortase A–mediated site‐specific conjugation. Additionally, alternative scFv‐F variants include tags such as an HA tag (for detection with anti‐HA antibody) and twin‐Strep‐TagII (for purification or detection with streptactin) (Fig. 2B).

### General Requirements

These protocols require a standard molecular biology setup (for cloning, *E. coli* growth, etc.) and experience with molecular biology methods. A fast protein liquid chromatography (FPLC) instrument with size‐exclusion chromatography (SEC) columns is required. The use of multichannel pipets is encouraged for working with a panel of scFvs. Sequence‐verified anti‐HS scFv constructs are required. The composition, preparation, and storage conditions for reagents are fully described in Reagents and Solutions. All solutions and equipment coming into contact with cells must be sterile, and proper sterile technique should be used accordingly. Gloves, goggles, and other protective equipment should be used as needed.

## Basic Protocol 1 EXPRESSION AND PURIFICATION OF ANTI‐HS scFvs FROM BACTERIAL EXPRESSION SYSTEMS

**Figure 3:**
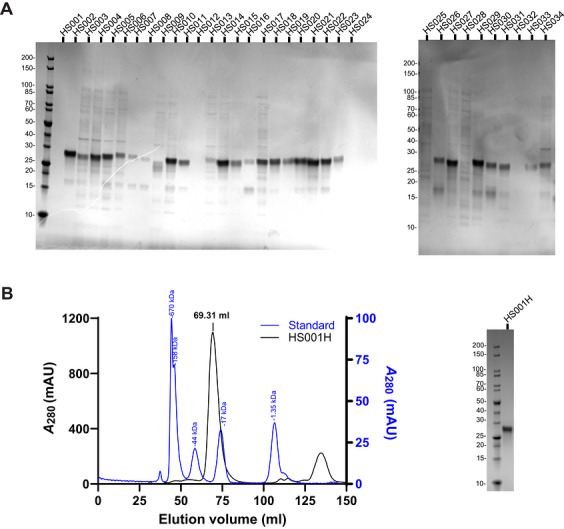
Expression and purification of anti‐HS scFvs from bacterial expression systems. (**A**) SDS‐PAGE for small‐scale expression screening of anti‐HS scFv‐H panel constructs. (**B**) SEC chromatogram and SDS‐PAGE of large‐scale expression and purification of HS001H. Chromatogram for SEC standards is normalized to 100% and represented in blue.

This protocol describes the workflow for expression and purification of the anti‐HS scFv‐H panel from the periplasmic space of *E. coli* (Fig. 1C). The constructs are first tested by small‐scale expression in a 96‐well format (Fig. 3A), followed by scaling up to larger volumes as needed (Fig. 3B). Because each of these scFvs is a distinct protein, yields vary between constructs (Table 2).

**Table 2:** Yields for Some Anti‐HS scFvs Purified in Our Facility

scFv	Approximate yields (mg/L culture)				
	H form	3×HA form	H_Avi	H_D2×st	F form
HS002	4.5	2.9	5.1	5.5	2.2
HS006	2.5	1.0	1.4	1.5	1.6
HS014	0.2	4.1	0.8	—	3.5
HS026	6.9	0.3	3.4	1.7	1.5
HS033	0.9	5.0	1.0	1.4	1.8
HS034	4.5	0.3	1.5	3.0	5.2

— Protein could not be purified.

### Materials

*E. coli* BL21‐CodonPlus (DE3)‐RIL competent cells (Agilent, cat. no. 230245)
Plasmid DNA
2×YT medium (see recipe):
  without antibiotics
  containing 34 µg/ml chloramphenicol, 50 µg/ml kanamycin, and 2% (w/v) glucose
  containing 50 µg/ml kanamycin only
1 M IPTG (see recipe)
scFv‐H resuspension buffer (see recipe)
50% (v/v) His60 slurry (see recipe), freshly prepared
His60 wash buffer (see recipe)
His60 elution buffer (see recipe)
4× Laemmli buffer (Bio‐Rad, cat. no. 1610747)
Dithiothreitol (DTT, Gold Bio, cat. no. DTT100)
Criterion XT precast gels, 4‐12% Bis‐Tris (Bio‐Rad, cat. no. 3450125)
Unstained protein standard (New England Biolabs, cat. no. P7717S)
SDS‐PAGE running buffer (see recipe)
SDS‐PAGE staining solution (see recipe)
LB agar plates (see recipe), 8 wells, with 34 µg/ml chloramphenicol, 50 µg/ml kanamycin, and 2% (w/v) glucose
10% (v/v) antifoam solution (see recipe)
scFv‐H SEC running buffer (see recipe)
Gel filtration standards (Bio‐Rad, cat. no. 1511901)

96‐well PCR plate (Thermo Fisher Scientific, cat. no. AB2396)
42°C heat block or thermocycler for 96‐well plate
96‐well 2‐ml deep‐well blocks (USA Scientific, cat. no. 1896‐2000), sterilized by autoclaving at 121°C for 15 min
AeraSeal breathable seals (Excel Scientific, cat. no. B100)
Bacterial shaking incubator at 37° and 22°C (e.g., Shel Lab S16R‐HS‐2 for small scale, New Brunswick Scientific I 26 for large scale)
Spectrophotometer (e.g., Stunner, Unchained Labs)
Bacterial plate incubator (e.g., Shel Lab S16R‐HS‐2) at 37°C
Plate shaker (e.g., Ika MTS 2/4) at 4°C
96‐well, 0.22‐µm filter plate (Thomson, cat. no. 931919)
Vacuum manifold for 96‐well filter plates (e.g., Sigma, cat. no. 57192‐U)
SDS‐PAGE gel running equipment (Bio‐Rad, cat. no. 1656019)
Gel imaging system (e.g., FluorChem, ProteinSimple)
250‐ml Erlenmeyer flasks (VWR, cat. no. 76531‐762)
2‐L baffled flasks (Genesis Pyrex, cat. no. 4446‐2L)
1‐L centrifuge bottles (Beckman, cat. no. C31597)
50‐ml centrifuge tubes (Fisher Scientific, cat. no. 14955239)
Rocker
20‐ml Econo‐Pac chromatography columns (Bio‐Rad, cat. no. 7321010)
0.45‐µm syringe filters (Genesee Scientific, cat. no. 25‐242)
FPLC system for protein purification (e.g., GE ÄKTA) equipped with HiLoad 16/600 Superdex 75‐pg column (Cytiva, cat. no. 28989333)
10‐kDa centrifugal concentrators (Amicon Ultra‐15, Millipore, cat. no. UFC901024)

#### Perform small‐scale expression test

##### Day 0: Transform cells and prepare inoculum

1 Cool a 96‐well PCR plate on ice. Thaw competent BL21‐CodonPlus (DE3)‐RIL cells on ice and dispense 10 µl into the necessary number of wells in the plate.
2 Add 1 µl (~50 ng) plasmid DNA and incubate on ice for 30 min.
3 Heat shock cells at 42°C for 45 s, then incubate on ice for 5 min.
4 Transfer cells to a 96‐well, 2‐ml deep‐well block containing 200 µl of 2×YT medium (no antibiotics). Seal with air‐permeable sealing sheet (e.g., AeraSeal) and incubate at 37°C and 750 rpm for 90 min.
  The optimal shaking speed may need to be determined for each instrument.
5 Add 900 µl of 2×YT medium containing 34 µg/ml chloramphenicol, 50 µg/ml kanamycin, and 2% (w/v) glucose. Incubate at 37°C and 750 rpm overnight.

###### Day 1: Prepare culture and induce expression

6 In a new 2‐ml deep‐well block, inoculate 1 ml fresh 2×YT medium containing 50 µg/ml kanamycin with 1% (v/v) overnight culture. Incubate at 37°C and 750 rpm until the OD_600_ is ∼0.6 (~2.5‐3 hr).
  When using other media (e.g., autoinduction media), the expression may have to be optimized for better yields. We have obtained good yields with 2×YT as a standard medium for all anti‐HS scFv bacterial expression constructs.
  We exclude chloramphenicol from the expression culture.
7 Induce cultures with 0.5 µl of 1 M IPTG (final 0.5 mM) and incubate at 22°C for 16 hr.

###### Day 2: Harvest cells and purify scFvs

8 Harvest cells by centrifuging 15 min at 4000 × *g*, 4°C. Discard the supernatant.
  Cell pellets can be stored at −80°C till further processing.
9 Resuspend cells with 500 µl cold scFv‐H resuspension buffer and incubate at 4°C and 600 rpm for 1 hr.
10 Centrifuge cells 15 min at 4000 × *g*, 4°C.
11 Transfer supernatant to a fresh 2‐ml block and add 100 µl freshly prepared His60 slurry. Incubate at 4°C and 600 rpm for 1 hr.
12 Transfer samples to filter plates attached to a vacuum pump.
13 Wash beads with His60 wash buffer 4 × 500 µl. Keep under vacuum pressure for 2 min to drain excess buffer.
14 Switch off the vacuum and transfer the filter plate to a fresh 96‐well PCR plate.
15 Add 50 µl His60 elution buffer and incubate at room temperature (RT) for 5 min.
16 Centrifuge plates 5 min at 500 × *g* to collect the eluted protein.
17 Transfer 15 µl eluant to a fresh 96‐well PCR plate.
18 Prepare 4× Laemmli buffer containing 50 mM DTT. Add 5 µl to each sample, mix, and incubate at 95°C for 5 min.
  Running the flowthroughs and washes from the His60 purification, at least the first time a protein is being purified, provides a good check to ensure the protocol works for the specific protein.
  DTT should be added to Laemmli buffer fresh before use.
19 Load 15 µl of each sample on a gel. Include 5 µl size standard in one lane.
20 Run gel at 175 V (constant voltage) for 40 min.
21 Remove gel, wash with H_2_O, and stain with SDS‐PAGE staining solution (Coomassie).
  Constructs that express protein in the small‐scale experiment can be used for large‐scale expression.

#### Perform large‐scale expression and purification

##### Day 0: Transform cells

22 Transform cells with 50 ng of the required constructs as described (see steps 1‐4).
23 Plate 20 µl transformation mix onto 8‐well LB agar plates with 34 µg/ml chloramphenicol, 50 µg/ml kanamycin, and 2% (w/v) glucose. Incubate overnight in a plate incubator at 37°C.
  Since the plates can be stored at 4°C for up to 2 weeks, we recommend transforming multiple constructs simultaneously.

###### Day 1: Prepare inoculum

24 Inoculate a single colony into a 250‐ml flask containing 50 ml of 2×YT medium with 34 µg/ml chloramphenicol, 50 µg/ml kanamycin, and 2% (w/v) glucose. Incubate overnight in a shaking incubator.

###### Day 2: Prepare large‐scale culture and induce expression

25 Prepare a 2‐L baffled flask with 500 ml of 2×YT medium containing 50 µg/ml kanamycin and 150 µl of 10% (v/v) antifoam solution. Inoculate with 10 ml overnight culture (2% v/v).
26 Incubate in a shaking incubator at 37°C and 175 rpm until the OD_600_ reaches 0.5 (~2.5‐3 hr).
  Because the culture can be frothy, we recommend using lower shaking speeds as well as an antifoam solution. We use Antifoam 204 at a final concentration of 0.003% (v/v) and a shaking speed of 175 rpm. If using a flat‐bottom flask, the shaking speed should be increased to improve aeration.
27 Adjust the incubator temperature to 22°C and let the culture equilibrate for 30 min.
28 Induce culture by adding 250 µl of 1 M IPTG (final 0.5 mM) and incubate at 22°C overnight (∼16 hr).

###### Day 3: Harvest cells and purify scFvs

29 Harvest cells by centrifuging 30 min at 4000 × *g*, 4°C, in a 1‐L centrifuge bottle. Discard the supernatant.
30 Transfer pellets to 50‐ml centrifuge tubes.
  The pellets can be stored at −80°C till further processing.
31 Resuspend cells in cold scFv‐H resuspension buffer and incubate at 4°C with gentle rocking for 1 hr.
  Cells should be resuspended in enough buffer to give a very dilute suspension (∼80‐100 ml per liter initial culture volume, depending on pellet size). This and all further steps should be carried out at 4°C using cold reagents.
32 Centrifuge cells 30 min at 4000 × *g*.
  This initial low‐speed centrifugation ensures removal of most cells from the suspension without lysing them. Cell lysis will result in release of cytoplasmic contents, thus reducing the purity of the initial sample, which may require additional purification steps.
33 Transfer supernatant to fresh 50‐ml centrifuge tubes and centrifuge 30 min at 12,000 × *g*.
  This higher‐speed centrifugation clarifies the supernatant of all cellular components.
34 Transfer supernatant to fresh 50‐ml centrifuge tubes.
35 Add 2 ml freshly prepared 50% (v/v) His60 slurry per liter of culture and incubate for 1 hr with gentle rocking.
  Immobilized metal affinity chromatography (IMAC) can be performed on an FPLC, but in our experience, the solution tends to be viscous, likely due to the high sucrose concentration as well as any genomic DNA that was released due to inadvertent cell lysis. This may clog prepacked columns. The viscosity can be reduced by adding DNase I or benzonase and diluting further with resuspension buffer (usually by four fold or more), but this will result in higher initial binding volumes.
36 Centrifuge 10 min at 500 × *g* to pellet the beads. Discard all but ∼3‐5 ml of supernatant.
37 Resuspend beads and transfer to an Econo‐Pac column.
38 Wash beads three times with 15 column volumes (CVs) of His60 wash buffer (3 × 5 ml).
39 Elute with 5 × 1 ml His60 elution buffer (with 5‐min incubations each time) into fresh 50‐ml centrifuge tubes.
  Alternatively, each elution can be collected separately and checked on an SDS‐PAGE gel before pooling the relevant samples and proceeding to SEC.
40 Pass the elute through a 0.45‐µm syringe filter to remove any precipitates.
41 Inject onto an SEC column pre‐equilibrated with scFv‐H SEC running buffer.
  The FPLC system and required SEC columns are generally stored in 20% (v/v) ethanol. To prepare them, pass H_2_O through the entire system and then equilibrate with scFv‐H running buffer. After the run is complete, pass H_2_O followed by 20% (v/v) ethanol. See Reagents and Solutions for H_2_O and ethanol that are appropriate for SEC.
42 Pool fractions that contain the protein and concentrate to 0.5‐1 mg/ml using a 10‐kDa centrifugal concentrator at 3000 × *g*.
  In our experience, some scFvs aggregate at higher concentrations.
43 Run an aliquot (∼1‐2 µg) on an SDS‐PAGE gel.
44 Aliquot, flash‐freeze, and store at −80°C till further use.
  For use, thaw aliquots on ice and keep at 4°C for up to 4‐6 weeks. Longer storage at 4°C is not recommended, as some scFvs lose activity after prolonged storage.
45 Run gel filtration standards according to manufacturer's instructions and compare to the results (Fig. 1B).

## Basic Protocol 2 EXPRESSION AND PURIFICATION OF ANTI‐HS scFvs FROM MAMMALIAN EXPRESSION SYSTEMS

**Figure 4:**
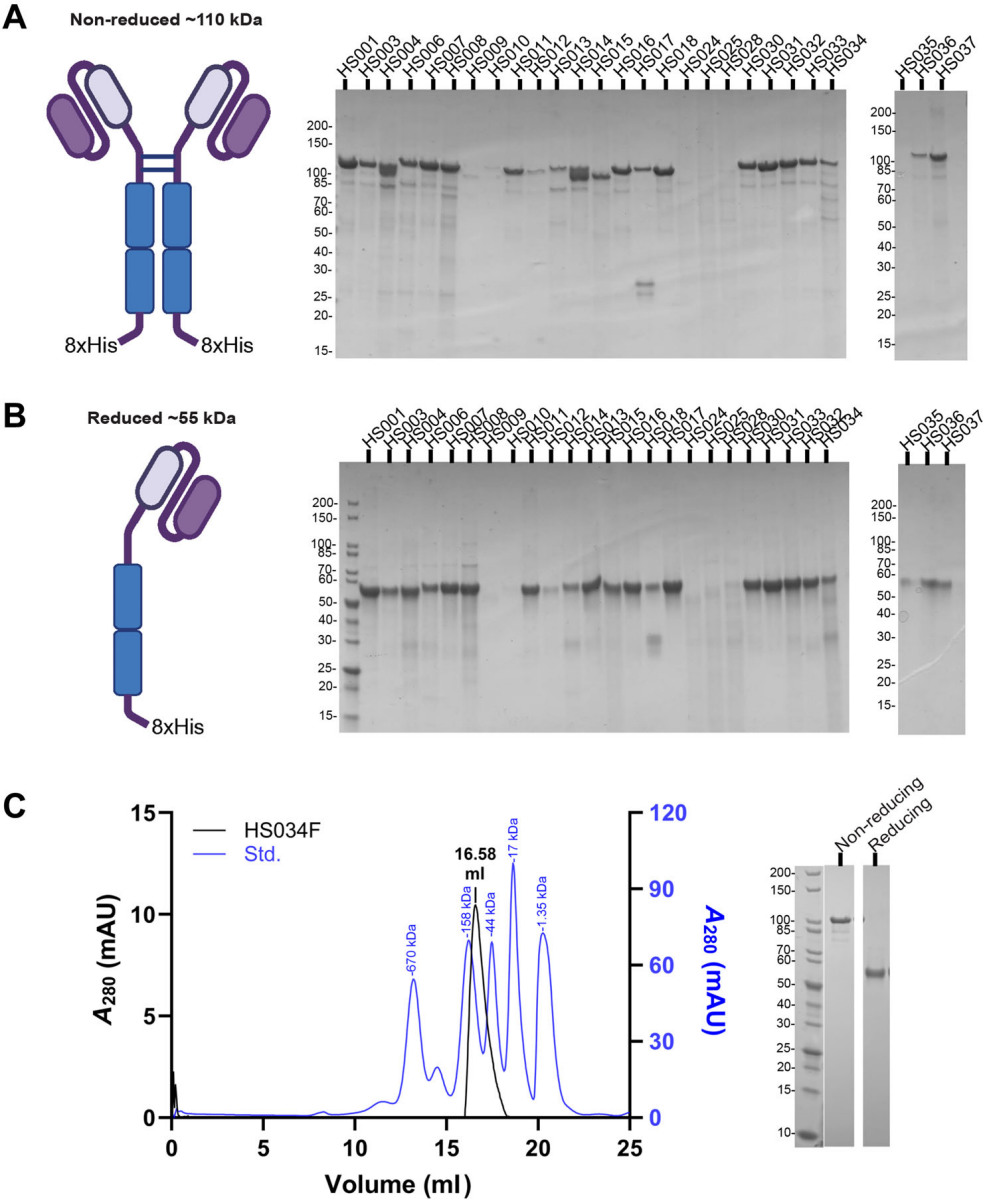
Expression and purification of anti‐HS scFvs from mammalian expression systems. (**A**,**B**) SDS‐PAGE for small‐scale expression screening of anti‐HS scFv‐F panel constructs under non‐reducing (A) and reducing (B) conditions. (**C**) Analytical SEC chromatogram and SDS‐PAGE of large‐scale expression and purification of HS034F. Chromatogram for SEC standards is normalized to 100% and represented in blue.

*NOTE*: For large‐scale expression, all steps must be carried out with minimal exposure to pyrogens (endotoxins) by using depyrogenated or pyrogen‐free glassware, plasticware, His60 beads, ÄKTA system and columns, and reagents. Glassware can be depyrogenated by incubating at 250°C for 3 hr. All surfaces that come into contact with samples, including the columns and components of the ÄKTA system, should be washed thoroughly with 0.5 M NaOH (Sigma, cat. no. 221465) in H_2_O.

This protocol describes the workflow for the expression and purification of endotoxin‐free anti‐HS scFvs using the ExpiCHO expression system, where proteins are secreted into the medium (Fig. 1C). Again, the constructs are first tested by small‐scale expression (Fig. 4A,B), then scaled up to larger volumes as needed (Fig. 4C) (Table 2). For small‐scale expression, we follow the manufacturer's standard titer protocol followed by protein A/G purification. For large‐scale expression, we follow the Thermo Fisher Scientific max titer protocol for a 100‐ml culture volume (see *ExpiCHO Expression System User Guide, 2018*), followed by His60 purification. The proteins exist as homodimers in solution (Fig. 4A), with the individual monomers (Fig. 4B) linked by disulfide bonds in the hinge region of the Fc section. The samples are run on an SDS‐PAGE gel with (Fig. 4B) and without (Fig. 4A) reducing agent to observe these two species.

### Materials

ExpiCHO Expression System (Thermo Fisher Scientific, cat. no. A29133)
Plasmid DNA prepared using endo‐free midi‐prep kit (e.g., Nucleobond Xtra Midi EF, Takara, cat. no. 740420.5)
OptiPRO SFM (Thermo Fisher Scientific, cat. no. 12309019)
Protein A/G magnetic beads (Pierce, cat. no. 88803)
1× PBS, pH 7.4, without calcium or magnesium (Gibco, cat. no. 70011‐04)
0.1 M glycine, pH 2.4 (VWR Life Science, cat. no. M103)
0.1 M Tris, pH 8.0 (Sigma, cat. no. T1503)
50% (v/v) His60 slurry (see recipe)
His60 wash buffer (see recipe)
His60 elution buffer (see recipe)
scFv‐F SEC running buffer (see recipe)
Glycerol (Sigma, cat. no. G7757)
Endotoxin test kit (Lonza chromogenic LAL assay, cat. no. 50‐650U)
Clear seal (Sigma, cat. no. Z369659‐100EA)

24‐well cell culture plates, sterile, non‐TC treated (Fisher Scientific, cat. no. CLS3738‐100EA)
1.5‐ml microcentrifuge tubes (Fisherbrand, cat. no. 05‐408‐129)
0.22‐µm syringe filters (UltraCruz, cat. no. sc‐395291)
Spectrophotometer (e.g., Stunner, Unchained labs)
Shaking incubator (e.g., Climo‐Shaker ISF‐4X) at 37°C, 110 rpm for culture flasks, 300 rpm for 24‐well plates, 8% CO_2_ and 85% humidity
Shaking incubator (e.g., Climo‐Shaker ISF‐4X) at 32°C, 115 rpm, 5% CO_2_ and 85% humidity
96‐well assay plate, V‐bottom, 500 µl (Axygen, cat. no. P‐96‐450V‐C‐S)
Magnetic bead separator rack for 96‐well plate (e.g., Beckman Coulter Agencourt, cat. no. A32782)
PCR plate (VWR, cat. no. 82006‐704)
500‐ml culture flasks, flat bottom (Genesee Scientific, cat. no. FPC0500S)
50‐ml centrifuge tubes (Genesee Scientific, cat. no. 21‐106)
500‐ml centrifuge bottles (Corning, cat. no. 431123)
Plate shaker (e.g., Ika MTS 2/4)
20‐ml Econo‐Pac chromatography columns (Bio‐Rad, cat. no. 7321010)
FPLC system for protein purification (e.g., GE ÄKTA) equipped with HiLoad 26/60 Superdex 200 pg and Superose 6 Increase 10/300 GL columns (GE, cat. nos. 17‐1071‐01 and 9‐0915‐96)
30‐kDa, 15‐ml centrifugal concentrators (Millipore, cat. no. UFC903024)

Additional reagents and equipment for SDS‐PAGE (see Basic Protocol 1)

#### Perform small‐scale expression test

##### Day 0: Transfect cells

*NOTE*: Always use cold reagents for transfection.

1 Dilute ExpiCHO cells to 6 × 10^6^ cells/ml in fresh medium.
  Cells should have a viability of >95%.
2 Aliquot 1 ml cells per well into a non‐treated 24‐well cell culture plate.
3 Dilute 1 µg plasmid DNA in 40 µl OptiPRO SFM in a 1.5‐ml centrifuge tube and mix by inverting. Filter using a 0.22‐µm syringe filter.
4 Mix the ExpiFectamine CHO reagent thoroughly by inverting the bottle. In a second 1.5‐ml tube, add 3.2 µl ExpiFectamine CHO reagent to 37 µl OptiPRO SFM and mix by inverting.
5 Add the ExpiFectamine mix to the DNA and invert to mix. Incubate at RT for 5 min.
6 Add the mix to the cells and incubate overnight in a 37°C shaking incubator.

###### Day 1: Add feed and enhancer

7 Add 6 µl ExpiFectamine CHO enhancer and 300 µl ExpiCHO feed to each well and return cells to the incubator for 6 days.

###### Day 7: Harvest cells and purify scFvs

8 Transfer 300 µl transfected cells to a fresh assay plate and centrifuge 2 min at 500 × *g*.
9 Transfer 250 µl of the supernatant to another fresh plate.
10 Add 10 µl protein A/G magnetic beads to each well.
11 Seal the plate with clear seal and incubate for 1 hr at RT with shaking (750 rpm on a plate shaker).
  All plate shaking steps should be carried out with some form of plate seal (e.g., clear seal) to prevent sample cross‐contamination.
12 Place on a 96‐well magnetic plate for 5 min. Remove supernatant using a Pasteur pipette connected to a vacuum supply.
13 Remove plate from the magnet and add 100 µl PBS/well. Shake on a plate shaker at 300 rpm for 1 min to resuspend beads in PBS.
14 Return plate to the magnet and remove PBS with a Pasteur pipette.
15 Repeat wash two more times.
16 Remove plate from the magnet and resuspend beads in 20 µl elution buffer (0.1 M glycine, pH 2.4). Incubate at RT for 5 min.
17 Return plate to the magnet and transfer eluates to a PCR plate.
18 Add 2 µl of 0.1 M Tris, pH 8.0.
19 Mix 15 µl eluate with 5 µl of 4× Laemmli buffer both with and without DTT. Heat samples for 5 min at 95°C.
20 Run on an SDS‐PAGE gel (see Basic Protocol 1, steps 19‐21).
  Results comparing samples under denaturing and non‐denaturing conditions are shown Fig. 4A,B.
  Constructs that express protein in the small‐scale experiment can be used for large‐scale expression.

#### Perform large‐scale expression

##### Day 0: Transfect cells

*NOTE*: Always use cold reagents for transfection.

21 Dilute ExpiCHO cells to 6 × 10^6^ cells/ml in fresh medium and dispense 100 ml per 500‐ml culture flask.
22 Dilute 100 µg plasmid DNA with 4 ml OptiPRO SFM in a 50‐ml centrifuge tube and mix by inverting. Filter through a 0.22‐µm syringe filter.
23 Mix the ExpiFectamine CHO reagent thoroughly by inverting the bottle. In a fresh tube, add 320 µl ExpiFectamine CHO reagent to 3.7 ml OptiPRO SFM and mix by inverting.
24 Add the ExpiFectamine mix to the DNA and mix by inverting. Incubate at RT for 5 min.
25 Add the mix to the cells and incubate overnight at 37°C in a shaking incubator.

###### Day 1: Add enhancer and feed

26 Add 600 µl ExpiFectamine CHO enhancer and 16 ml ExpiCHO feed and incubate for 4 days in a 32°C shaking incubator at 115 rpm.

###### Day 5: Add feed

27 Add 16 ml ExpiCHO feed and return flasks to the incubator for 7 days.

###### Day 12: Harvest cells and purify scFvs

28 Harvest cells by centrifuging 15 min at 500 × *g* in 500‐ml centrifuge bottles.
29 Transfer supernatant to fresh 500‐ml centrifuge bottles and centrifuge 15 min at 2000 × *g*.
30 Add 2 ml freshly prepared 50% (v/v) His60 slurry to the supernatant and incubate 1 hr at RT with shaking.
31 Load the suspension onto a 20‐ml Econo‐Pac column.
32 Wash beads three times with 15 column volumes (CVs) of His60 wash buffer (3 × 5 ml).
33 Elute with 5 × 1 ml His60 elution buffer (with 5‐min incubations each time) into fresh 50‐ml centrifuge tubes.
  Alternatively, each elution can be collected separately and checked for protein expression on an SDS‐PAGE gel before pooling the relevant samples and proceeding to SEC.
34 Pass the elute through a 0.22‐µm syringe filter to remove any precipitates.
35 Inject onto an SEC column pre‐equilibrated with scFv‐F SEC running buffer.
  The FPLC system and required SEC columns are generally stored in 20% (v/v) ethanol. To prepare them for endotoxin‐free scFv‐F preparation, pass H_2_O and 0.5 M NaOH through the entire system, then equilibrate with scFv‐H running buffer. After the run is complete, pass 0.5 M NaOH and H_2_O followed by 20% (v/v) ethanol. See Reagents and Solutions for NaOH and H_2_O that are appropriate for SEC.
*CAUTION*: From this step onwards, handle samples in a biosafety cabinet to minimize exposure to contaminants.
36 Pool fractions that contain protein and concentrate to 0.5‐1 mg/ml using a 30‐kDa centrifugal concentrator at 3000 × *g*.
  In our experience, some scFvs aggregate at higher concentrations.
37 Run an aliquot (∼1‐2 µg) on an analytical SEC column and SDS‐PAGE gel.
38 Add glycerol to a final concentration of 10% (v/v).
39 Sterile filter the samples through a 0.22‐µm syringe filter.
40 Measure endotoxin levels using a specialized kit if samples will be used in endotoxin‐sensitive downstream applications.
41 Aliquot, flash‐freeze, and store at −80°C till further use.
  For use, thaw aliquots on ice and keep at 4°C for up to 4‐6 weeks. Longer storage at 4°C is not recommended, as some scFvs lose activity after prolonged storage.

## Basic Protocol 3 SORTASE‐MEDIATED SITE‐SPECIFIC LABELING OF ANTI‐HS scFvs

**Figure 5:**
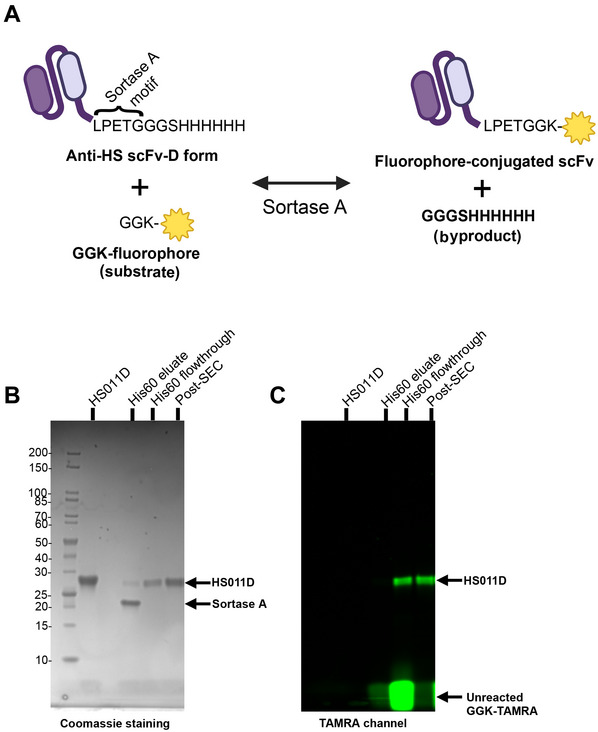
Sortase‐mediated site‐specific labeling of anti‐HS scFvs. (**A**) Schematic of site‐specific sortase A–mediated fluorophore conjugation of an anti‐HS scFv. (**B**,**C**) Conjugation of HS011D with fluorophore GGK‐TAMRA by SDS‐PAGE. The gel was visualized on a FluorChem instrument with white light/Coomassie staining (B) and with a green filter (537/35 nm) (C). Sortagging efficiency was calculated to be >95% (based on near absence of scFv in the His60 elute fraction). Degree of labeling was calculated to be >90% (based on absorbance values).

Site‐specific labeling techniques provide convenient and controlled labeling strategies for producing antibodies conjugated with specific moieties such as fluorophores, DNA oligos, and other chemical modifications. Sortase A is a transpeptidase that can specifically label the amino acid sequence LPxTG, where x is any residue (Theile et al., 2013). This protocol describes sortase A–mediated site‐specific labeling of anti‐HS scFvs with a fluorophore (Fig. 5). A protocol for FACS analyses using fluorophore‐conjugated anti‐HS scFvs is described in Alternate Protocol 2.

### Materials

Anti‐HS scFv with sortase motif (D or D2×st)
1 M HEPES, pH 7.4
5 M NaCl
GGK‐TAMRA (substrate, custom‐ordered from GenScript)
Sortase A 7M enzyme (produced from Addgene plasmid 51141 according to Theile et al., 2013)
His60 wash buffer (see recipe)
50% (v/v) His60 slurry (see recipe)
scFv‐H SEC running buffer (see recipe)
His60 elution buffer (see recipe)

0.22‐µm centrifugal filters (Millipore, cat. no. UFC30GV0S)
10‐kDa, 500‐µl centrifugal concentrators (Millipore, cat. no. UFC501024)
FPLC system for protein purification (e.g., GE ÄKTA) equipped with Superose 6 Increase 10/300 GL column (GE, cat. no. 9‐0915‐96)
Spectrophotometer (e.g., Stunner, Unchained labs)

Additional reagents and equipment for SDS‐PAGE (see Basic Protocol 1)

#### Label scFv

1 Exchange buffer in the scFv of interest to 20 mM HEPES, pH 7.4, with 150 mM NaCl (to remove glycerol) and concentrate to >75 µM.
  Care must be taken to avoid over‐concentrating, as some scFvs may precipitate at higher concentrations.
2 Set up a 100‐µl labeling reaction as follows:
  2 µl 1 M HEPES, pH 7.4 (final 20 mM)
  3 µl 5 M NaCl (final 120 mM)
  38.5 µl 104 µM scFv (final 40 µM)
  10 µl 50 mM GGK‐TAMRA (final 2 mM)
  1.7 µl 4.4 mM sortase A (final 76 µM)
  44.8 µl H_2_O
3 Incubate samples at 37°C for 1 hr.

#### Remove unmodified scFv

4 Dilute sample with 400 µl His60 wash buffer.
5 Add 100 µl freshly prepared 50% (v/v) His60 slurry and incubate overnight at 4°C.
6 Separate flowthrough from beads using a centrifugal filter at 500 × *g* for 2 min. Collect flowthrough and concentrate to ~100 µl using a centrifugal concentrator at 11,000 × *g* for 2‐5 min.
  This fraction contains the fluorophore‐conjugated scFv and unmodified substrate.
7 Perform a final SEC with scFv‐H or scFv‐F SEC running buffer to remove unlabeled substrate.
  Use scFv‐H running buffer for proteins from bacterial expression systems and scFv‐F running buffer for proteins from mammalian expression systems.
  The FPLC system and SEC columns are generally stored in 20% (v/v) ethanol. To prepare them, first pass H_2_O through the entire system and then equilibrate with scFv‐H (or scFv‐F) running buffer. After the run is complete, pass H_2_O followed by 20% (v/v) ethanol. See Reagents and Solutions for H_2_O and ethanol that are appropriate for SEC.
8 Pool the fractions that contain labeled scFv and concentrate to 1 mg/ml using a centrifugal concentrator.
9 Store up to 2 weeks at 4°C.

#### Analyze labeled scFv

10 Measure absorbance at 280 nm.
11 Calculate the protein concentration and degree of labeling as follows:
$$\mathrm{Protein}\;\mathrm{concentration}\left(M\right)=\left(A_{280}-\left[A_{\max}\times \mathrm{CF}\right]\right)\times \left(\mathrm{dilution}\;\mathrm{factor}/\varepsilon \right)$$
  where *A*_280_ is the absorbance of the labeled sample at 280 nm, *A*_max_ is the absorbance of dye solution measured at the wavelength maximum for the specific dye, CF is the correction factor for the dye at 280 nm, and ε is the molar extinction coefficient of the protein (in M^−1^ cm^−1^).
$$\begin{array}{ccc} & & \mathrm{Degree}\;\mathrm{of}\;\mathrm{labeling}\left(\mathrm{mol}\;\mathrm{dye}/\mathrm{mol}\;\mathrm{protein}\right)\\ & & \;=\left(A_{\max}\times \mathrm{DF}\right)/\left(\varepsilon^{\prime }\times \mathrm{protein}\;\mathrm{concentration}\right) \end{array}$$
  where DF is the dilution factor and ε′ is the molar extinction coefficient of the dye (in M^−1^ cm^−^^1^).
  Additional information for these calculations can be found in the Thermo Scientific (2011) publication “Tech Tip #31. Calculate dye:protein (F/P) molar ratios.”
12 Elute the bound material from the His60 resin using His60 elution buffer.
  This fraction will contain sortase A and unreacted scFv, both of which contain His tags.
13 Run all samples on an SDS‐PAGE gel.
  The eluate lane will contain sortase A and any unlabeled scFv, the His60 flowthrough lane will contain labeled scFv and unreacted GGK‐TAMRA, and the post‐SEC lane will contain just the labeled scFv (Fig. 5B,C).

## Basic Protocol 4 *IN VITRO* HS DETECTION BY ELISA

**Figure 6:**
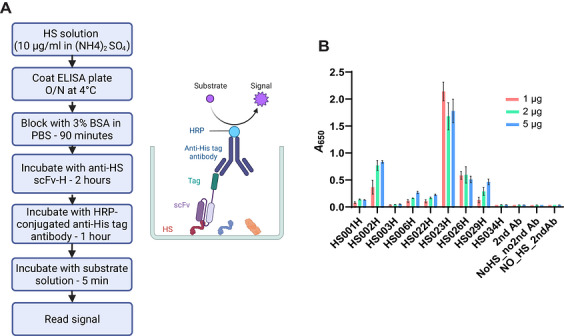
Use of anti‐HS scFvs as detection reagents for ELISA. (**A**) Schematic of workflow for ELISA experiments. (**B**) Representative ELISA data from duplicate samples. HS from bovine kidney was coated onto polystyrene plates and detected using anti‐HS scFv‐H forms as primary antibody and HRP‐conjugated anti‐His antibody as the secondary antibody. The plot shows mean *A*_650_ signals (solid bars) with range (error bars) of data collected from two separate wells (*n* = 2). HS034 is a negative control scFv and has no known epitope.

ELISAs have been used extensively to screen anti‐HS scFvs (Smits et al., 2006). Here, we describe a workflow for the use of anti‐HS scFv for HS detection via ELISA (Fig. 6A). As an example, we use an anti‐His antibody for detection (Fig. 6B). We have also successfully used with other tags, such as anti‐HA and anti–human IgG. Similarly, heparin can be immobilized instead of bovine kidney HS.

### Materials

10 µg/ml HS from bovine kidney (sodium salt, Sigma, cat. no. H7640) in 4.1 M ammonium sulfate solution (Fisher Scientific, cat. no. BP212‐212)
ELISA wash buffer (see recipe)
Blocking buffer (see recipe)
scFv‐H SEC running buffer (see recipe)
PBST (see recipe)
HRP‐conjugated anti‐His (Sigma‐Aldrich, cat. no. A7058) diluted 1:3000 in blocking buffer
Substrate solution (Thermo Fisher, 1‐Step Ultra TMB‐ELISA, cat. no. 34028)

96‐well flat‐bottom plate, polystyrene, non‐treated (Corning, cat. no. 3370)
Clear seal (Sigma‐Aldrich, cat. no. Z369659)
Plate reader capable of measuring absorbance at 650 nm

1 Coat wells of an ELISA plate with 100 µl of 10 µg/ml bovine kidney HS in 4.1 M (NH_4_)_2_SO_4_.
  When laying out the plate, include controls for (1) no scFv (with HS and secondary antibody), (2) no scFv or HS (with secondary antibody), and (3) no scFv, HS, or secondary antibody (see Fig. 6B).
2 Seal the plate to prevent evaporation and incubate overnight at 4°C.
3 Discard solution and wash plate six times with 350 µl ELISA wash buffer.
4 Block with 350 µl blocking buffer for 90 min at RT.
5 During this time, prepare the anti‐HS scFv reagents. First, add 1, 2, or 5 µg of each scFv to scFv‐H or scFv‐F SEC buffer to give a total volume of 20 µl. Then, add 80 µl blocking buffer to bring to 100 µl.
  Use scFv‐H running buffer for proteins from bacterial expression systems or scFv‐F for proteins from mammalian expression systems.
6 Discard blocking solution and add 100 µl scFv solutions to the appropriate wells. Incubate for 2 hr at RT.
7 Discard solution and wash plate six times with 350 µl PBST.
8 Add 100 µl anti‐His‐HRP secondary antibody (1:3000 in blocking buffer) to the appropriate wells and incubate for 1 hr at RT in the dark.
9 Discard solution and wash plate six times with 350 µl PBST.
10 Add 50 µl substrate solution to each well and incubate at RT in the dark until color development is clearly visible (∼5‐10 min).
11 Measure absorbance at 650 nm on a plate reader.

## Basic Protocol 5 *IN VITRO* HS DETECTION BY FLOW CYTOMETRY USING INDIRECT LABELING IN LIVE JURKAT E6‐1 CELLS

**Figure 7:**
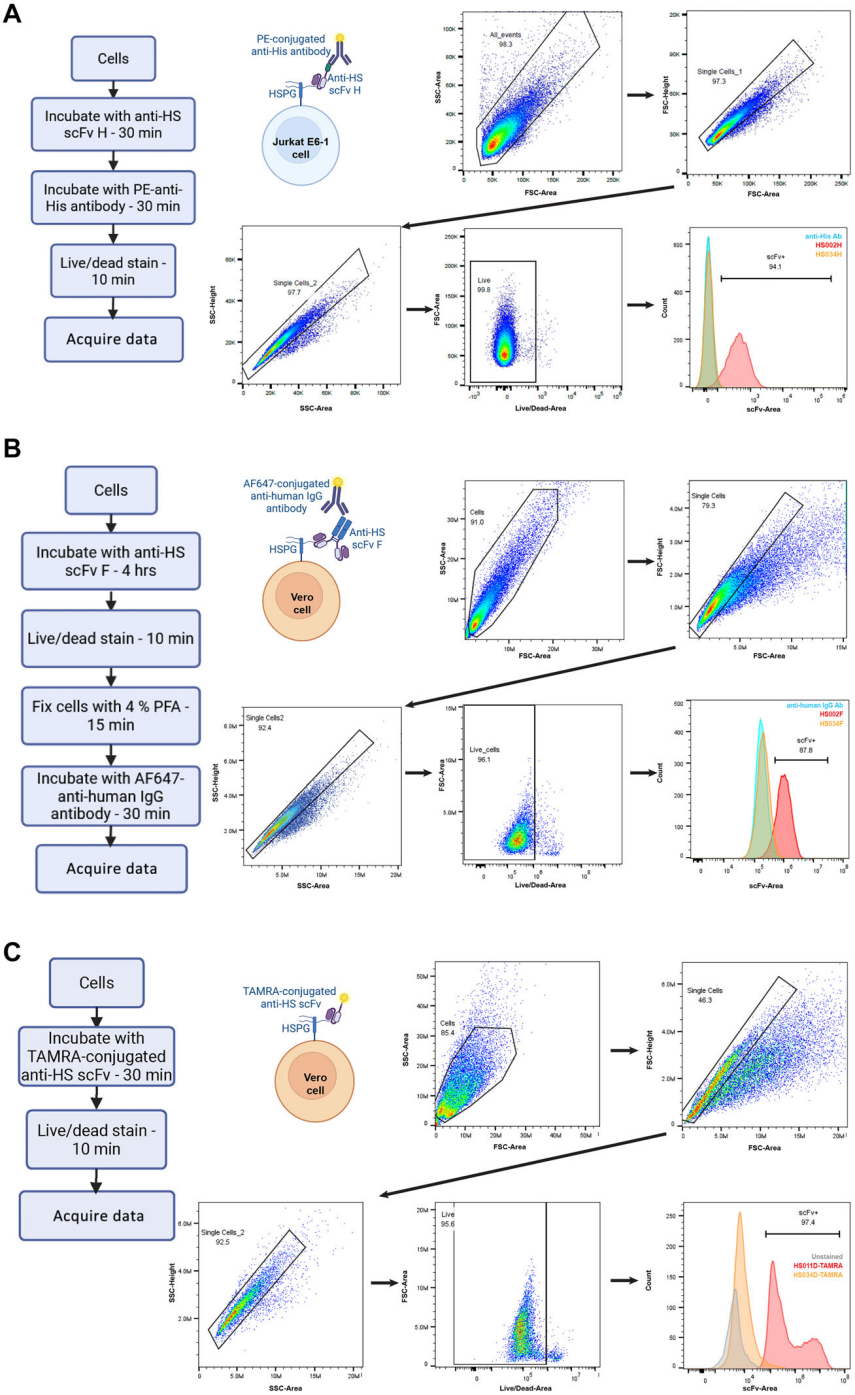
Use of anti‐HS scFvs as staining reagents for flow cytometry. (**A‐C**) Workflow, gating strategy, and representative histograms for FACS analyses of (**A**) live Jurkat E6‐1 cells stained with anti‐HS scFv‐H HS001 and PE‐conjugated anti‐His antibody (data collected on Sony SA3800 spectral analyzer); (**B**) Vero cells stained with anti‐HS scFv‐H HS001, then fixed and detected using AlexaFluor647‐conjugated anti‐human IgG antibody (data collected on Novocyte Quanteon in AlexaFluor647 channel); and (**C**) live Vero cells stained with TAMRA‐conjugated anti‐HS scFvs (data collected on Novocyte Quanteon in the PE/TAMRA channel). HS034 is a negative control scFv and has no known epitope. scFv staining observed in live, single‐cell populations.

In this section, we describe three different workflows and associated protocols for staining cells with the anti‐HS scFv panel for flow cytometry experiments (Fig. 7) to demonstrate the adaptability of the panel for different applications. This protocol describes indirect labeling of live Jurkat E6‐1 cells using anti‐HIS scFv forms and a fluorophore‐conjugated secondary anti‐His antibody (Fig. 7A). Alternate Protocol 1 describes indirect labeling of fixed Vero cells (Fig. 7B) and Alternate Protocol 2 describes direct labeling of live Vero cells using fluorophore‐conjugated anti‐HS scFvs (Fig. 7C).

### Materials

Jurkat E6‐1 cells (ATCC, TIB‐152) maintained in 125‐ml flat‐bottomed flasks per ATCC recommendations, cultured in IMDM with GlutaMAX (Gibco, cat. no. 31980097) and 10% FBS (Sigma, cat. no. 12306C)
FACS buffer (see recipe)
0.4% (w/v) trypan blue (Invitrogen, cat. no. T10282)
Primary antibodies: 2 µg anti‐HS scFv‐H forms in 50 µl FACS buffer
Secondary antibody: anti‐His‐PE (Miltenyi, cat. no. 130‐120‐718) diluted 1:200 in FACS buffer
Live/dead stain: Fixable Viability Stain 780 (BD, cat. no. 565388) diluted 1:1000 in 1× PBS, pH 7.4, without calcium or magnesium (Gibco, cat. no. 70011‐04)

50‐ml centrifuge tubes (Genesee Scientific, cat. no. 21‐106)
Automated cell counter and slides (e.g., Invitrogen Countess Automated Cell Counter, cat. nos. C10281 and C10228)
96‐well conical‐bottom, non‐treated, polystyrene plates (Nunc, cat. no. 249570) and lids (Corning, cat. no. 3098)
Clear seal (Sigma‐Aldrich, cat. no. Z369659)
Plate shaker (e.g., Ika MTS 2/4)
Cytometer (e.g., Sony SA3800 spectral analyzer or Agilent Novocyte Quanteon)

#### Harvest cells

1 Transfer Jurkat E6‐1 cells to a 50‐ml centrifuge tube and centrifuge 3 min at 500 × *g*. Decant supernatant.
  Steps 1‐13 are performed at 4°C with cold reagents.
2 Resuspend pellet in FACS buffer and count total and live cells in an automated cell counter using trypan blue stain.
3 Dilute cells to 1 × 10^6^ cells/ml in FACS buffer.

#### Label cells

4 Dispense 50 µl (50,000 cells) into the wells of a 96‐well conical‐bottom microplate.
  Include single‐color control wells for unmixing/compensation purposes.
  All plate shaking steps should be carried out with some form of plate seal (e.g., clear seal) to prevent sample cross‐contamination.
5 Centrifuge 3 min at 500 × *g* and discard the supernatant.
6 Add primary antibody solutions containing 2 µg anti‐HS scFv in 50 µl FACS buffer.
7 Resuspend cells for 3 min on a plate shaker at 600 rpm, then reduce speed to 300 rpm and incubate for 30 min.
8 Centrifuge 3 min at 500 × *g* and discard the supernatant.
9 Wash cells by resuspending in 150 µl FACS buffer for 3 min on a plate shaker at 600 rpm.
10 Centrifuge 3 min at 500 × *g* and discard the supernatant.
  In all subsequent steps, the samples must be protected from light to prevent photobleaching.
11 Add 50 µl conjugated secondary antibody to the appropriate wells.
12 Resuspend cells for 3 min on a plate shaker at 600 rpm, then reduce speed to 300 rpm and incubate for 30 min.
13 Wash as in steps 8‐10.
  All subsequent steps are carried out at RT with RT reagents.
14 Resuspend cells in 50 µl live/dead stain and incubate for 10 min at RT on a plate shaker at 600 rpm.
15 Wash as in steps 8‐10.
16 Resuspend in 100 µl FACS buffer for 3 min on a plate shaker at 600 rpm.
17 Collect data on a cytometer.

## Alternate Protocol 1 *IN VITRO* HS DETECTION BY FLOW CYTOMETRY USING INDIRECT LABELING IN FIXED VERO CELLS

This protocol describes staining of Vero cells using anti‐HS scFv forms and a fluorophore‐conjugated secondary anti‐His antibody with fixation between the two labeling steps (Fig. 7B). The harvesting method for adherent cells depends on the cell type and how strongly the cells adhere to the culture plate. Weakly adherent cells such as HEK293 cells can be harvested by incubating with PBS + 5 mM EDTA followed by gentle pipetting to give a single‐cell suspension. For more adherent cells such as Vero or HaCaT cells, enzymatic dissociation (with TrypLE or Trypsin) may be required, as described below. Care must be taken to not incubate cells for long periods in enzymatic solutions, as this may result in cleavage and release of cell‐surface proteins, which can affect HS detection.

### Materials

Vero cells (ATCC, CCL‐81) maintained in 10‐cm TC‐treated cell culture dishes as per ATCC recommendations, cultured in DMEM with l‐glutamine, glucose, and sodium pyruvate (Corning, cat. no. 10013CV) supplemented with 10% FBS (Sigma, cat. no. 12306C)
1× PBS pH 7.4 without calcium or magnesium (Gibco, cat. no. 70011‐04)
TrypLE Express (Gibco, cat. no. 12604013)
0.4% (w/v) trypan blue (Invitrogen, cat. no. T10282)
Primary antibodies: 5 µg anti‐HS scFv‐H forms in 50 µl DMEM without FBS
Live/dead stain: Fixable Viability Stain 780 (BD, cat. no. 565388) diluted 1:1000 in 1× PBS
4% (w/v) PFA in 1× PBS (ChemCruz, cat. no. sc‐281692) at 4°C
FACS buffer (see recipe)
Secondary antibody: anti‐His‐PE (Miltenyi, cat. no. 130‐120‐718) diluted 1:200 in FACS buffer

50‐ml centrifuge tubes (Genesee Scientific, cat. no. 21‐106)
Automated cell counter and slides (e.g., Invitrogen Countess Automated Cell Counter, cat. nos. C10281 and C10228)
96‐well round‐bottom, non‐treated, polystyrene plates with lids (Falcon, cat. no. 351177)
Clear seal (Sigma‐Aldrich, cat. no. Z369659)
96‐well conical‐bottom, non‐treated, polystyrene plates (Nunc, cat. no. 249570) and lids (Corning, cat. no. 3098)
Plate rocker (e.g., Stovall Belly Dancer Orbital Lab Shaker)
Plate shaker (e.g., Ika MTS 2/4)
Cytometer (e.g., Sony SA3800 spectral analyzer or Agilent Novocyte Quanteon)

#### Harvest cells

1 Remove spent medium from a 10‐cm plate of Vero cells (~80‐90% confluent) and rinse plate gently with PBS.
2 Incubate cells at RT for ∼10‐15 min with 1 ml TrypLE until they are dissociated.
  All subsequent steps are carried out at 4°C using cold reagents, and DMEM is used without FBS.
3 Add 5 ml fresh DMEM and transfer to 50‐ml centrifuge tubes.
4 Centrifuge 4 min at 500 × *g*. Discard supernatant.
5 Resuspend pellet in fresh DMEM and count total and live cells in an automated cell counter using trypan blue stain.
6 Dilute to 2 × 10^6^ cells/ml in fresh DMEM.

#### Label cells

7 Dispense 50 µl cells into a 96‐well round‐bottom plate (100,000 cells/well).
  Include single‐color control wells for unmixing/compensation purposes.
8 Add primary antibody solutions containing 5 µg anti‐HS scFv in 50 µl DMEM.
9 Incubate for 4 hr in a rocker.
  All plate shaking steps should be carried out with some form of plate seal (e.g., clear seal) to prevent sample cross‐contamination.
10 Centrifuge 5 min at 500 × g and discard the supernatant.
11 Wash by resuspending cells in 200 µl PBS. Centrifuge 5 min at 500 × *g* and discard the supernatant.
12 Repeat step 11.
13 Resuspend cells in 50 µl live/dead stain and incubate for 10 min at RT on a plate shaker at 600 rpm.
  In this experiment, live/dead stain must be added before fixing the cells.
14 Centrifuge at 500 × *g* for 5 min and discard the supernatant.
15 Wash twice as in step 11.
16 Fix cells with 100 µl cold 4% PFA for 15 min at RT.
17 Wash twice as in step 11.
18 Resuspend cells in 100 µl FACS buffer.
  In all subsequent steps, samples must be protected from light to prevent photobleaching.
  Fixed cells can be stored overnight at 4°C to provide a stopping point.
19 Transfer samples to a 96‐well conical‐bottom plate.
20 Centrifuge 5 min at 500 × *g* and discard the supernatant.
21 Add 50 µl conjugated secondary antibody to the appropriate wells.
22 Resuspend cells for 3 min on a plate shaker at 600 rpm, then reduce speed to 300 rpm and incubate for 30 min.
23 Centrifuge at 500 × *g* for 3 min and discard the supernatant.
24 Wash by resuspending cells in 150 µl FACS buffer and shaking on a plate shaker for 3 min at 600 rpm. Centrifuge at 500 × *g* for 3 min and discard the supernatant.
25 Repeat step 24.
26 Resuspend in 100 µl FACS buffer for 3 min on a plate shaker at 600 rpm.
27 Collect data on a cytometer.

## Alternate Protocol 2 *IN VITRO* HS DETECTION BY FLOW CYTOMETRY USING DIRECT LABELING IN LIVE VERO CELLS

This protocol describes direct labeling in live Vero cells using fluorophore‐conjugated anti‐HS scFvs (Fig. 7C). The labeled scFvs are prepared as described in Basic Protocol 3.

### Materials

Vero cells (ATCC, CCL‐81) maintained in 10‐cm TC‐treated cell culture dishes as per ATCC recommendations, cultured in VP‐SFM (Thermo Fisher Scientific, cat. no. 11681020) with 4 mM GlutaMAX (Thermo Fisher Scientific, cat. no. 35050061)
1× PBS pH 7.4 without calcium or magnesium (Gibco, cat. no. 70011‐04)
TrypLE Express (Gibco, cat. no. 12604013)
FACS buffer (see recipe)
0.4% (w/v) trypan blue (Invitrogen, cat. no. T10282)
Primary antibodies: 2 µg fluorophore‐conjugated anti‐HS‐scFv forms in 50 µl FACS buffer
Live/dead stain: Fixable Viability Stain 780 (BD, cat. no. 565388) diluted 1:1000 in 1× PBS

50‐ml centrifuge tubes (Genesee Scientific, cat. no. 21‐106)
Automated cell counter and slides (e.g., Invitrogen Countess Automated Cell Counter, cat. nos. C10281 and C10228)
96‐well conical‐bottom, non‐treated, polystyrene plates (Nunc, cat. no. 249570) and lids (Corning, cat. no. 3098)
Clear seal (Sigma‐Aldrich, cat. no. Z369659)
Plate shaker (e.g., Ika MTS 2/4)
Cytometer (e.g., Sony SA3800 spectral analyzer or Agilent Novocyte Quanteon)

#### Harvest cells

1 Remove spent medium from a 10‐cm plate of Vero cells and rinse plate gently with PBS.
2 Incubate cells at RT for ∼10‐15 min with 1 ml TrypLE until they are dissociated.
3 Add 5 ml fresh VP‐SFM and transfer cells to 50‐ml centrifuge tubes.
4 Centrifuge 4 min at 500 × *g* and discard supernatant.
  All subsequent steps are performed with at 4°C using cold reagents.
5 Resuspend pellet in FACS buffer and count total and live cells in an automated cell counter using trypan blue stain.
6 Dilute to 1 × 10^6^ cells/ml in FACS buffer.

#### Label cells

7 Dispense 100 µl into a 96‐well conical‐bottom microplate (100,000 cells/well).
  Include single‐color control wells for unmixing/compensation purposes.
8 Centrifuge 3 min at 500 × *g* and discard the supernatant.
  In all subsequent steps, samples must be protected from light to prevent photobleaching.
9 Add primary antibody solutions containing 2 µg fluorophore‐conjugated anti‐HS‐scFv in 50 µl FACS buffer.
10 Resuspend cells for 3 min on a plate shaker at 600 rpm, then reduce the speed to 300 rpm and incubate for 30 min.
  All plate shaking steps should be carried out with some form of plate seal (e.g., clear seal) to prevent sample cross‐contamination.
11 Centrifuge 30 min at 500 × *g* and discard the supernatant.
12 Wash by resuspending cells in 150 µl FACS buffer and shaking on a plate shaker for 3 min at 600 rpm.
13 Centrifuge 3 min at 500 × *g* and discard the supernatant.
  The subsequent steps are carried out at RT with RT reagents.
14 Resuspend cells in 50 µl live/dead stain and incubate for 10 min on a plate shaker at 600 rpm.
15 Wash as in steps 11‐13.
16 Resuspend in 100 µl FACS buffer for 3 min on a plate shaker at 600 rpm.
17 Collect data on a cytometer.

## Basic Protocol 6 *IN VITRO* HS DETECTION BY IMMUNOFLUORESCENCE IN FIXED ADHERENT CELLS

**Figure 8:**
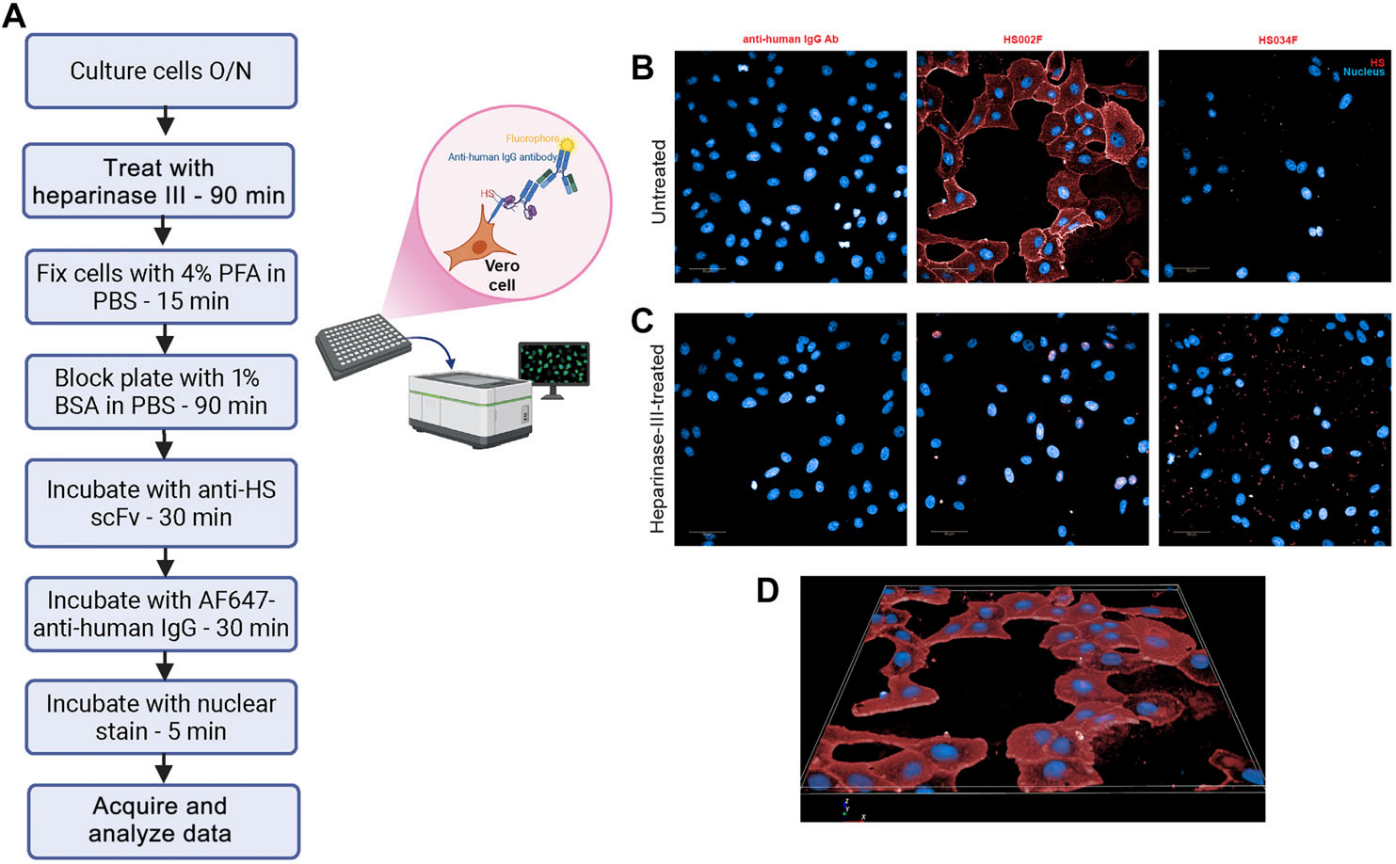
Use of anti‐HS scFvs as staining reagents for immunofluorescence staining of adherent cells. (**A**) Workflow. (**B‐D**) Representative 40× confocal images acquired with Operetta CLS 4 LED system. For each field, an image stack of 15 layers (0.5 µm separation between layers) was acquired with a 40× water objective. Scale bars are 50 µm. Nuclei (blue) detected with Hoechst 33258 channel (ex. 355‐385 nm; em. 430‐500 nm). scFv detected with AlexaFluor 647 channel (filters: ex.: 615‐645 nm; em.: 655‐760 nm) represented in red. All images were collected with the same acquisition settings and are shown using the same display settings. (**B,C**) Left to right: secondary antibody control, HS002F, and HS034F (negative control). Projection view of 5 layers for (B) anti‐HS scFv staining and (C) anti‐HS scFv staining after heparinase III treatment. (**D**) 3D projection of HS002 from (B) reconstructed with all 12 layers. HS034 is a negative control scFv and has no known epitope.

In this section, we describe two workflows and associated protocols for staining cells with anti‐HS scFv‐F forms for immunofluorescence studies to demonstrate the adaptability of the anti‐HS scFv panel across different applications. This protocol describes staining of fixed adherent cells and Alternate Protocol 3 describes staining of live suspension cells followed by fixation. Adherent cells are grown in immunofluorescence‐compatible 96‐well plates and stained directly on the plate, allowing better visualization of cellular morphology. The seeded cells are grown for ∼20 hr, then treated with heparinase III (to show that the scFvs specifically recognizes HS) and stained with anti‐HS scFv‐F forms as the primary antibody followed by fluorophore‐conjugated anti–human IgG as the secondary antibody (Fig. 8A). Cells are then stained with Hoechst 33258 nuclear stain and imaged (Fig. 8B‐D).

### Materials

Vero cells (ATCC, CCL‐81) maintained in 10‐cm TC‐treated cell culture dishes as per ATCC recommendations, cultured in VP‐SFM (Thermo Fisher Scientific, cat. no. 11681020) + 4 mM GlutaMAX supplement (Thermo Fisher Scientific, cat. no. 35050061)
1× PBS, pH 7.4, without calcium or magnesium (Gibco, cat. no. 70011‐04)
TrypLE Express (Gibco, cat. no. 12604013)
0.4% (w/v) trypan blue (Invitrogen, cat. no. T10282)
Heparinase III (lab‐made, New England Biolabs, or Sigma)
1× DPBS pH 7.2 with calcium and magnesium (Gibco, cat. no. 14040133)
4% (w/v) paraformaldehyde (PFA) in 1× PBS (ChemCruz, cat. no. sc‐281692) at 4°C
Immunofluorescence staining buffer (see recipe)
Primary antibodies: 2 µg anti‐HS scFv‐F forms in 50 µl staining buffer
Secondary antibody: AlexaFluor‐647‐conjugated anti–human IgG (Invitrogen) diluted 1:250 in staining buffer
10 µg/ml Hoechst 33258: 10 mg/ml stock (Molecular Probes, cat. no. R11496) diluted 1:1000 in PBS

50‐ml centrifuge tubes (Genesee Scientific, cat. no. 21‐106)
Automated cell counter and slides (e.g., Invitrogen Countess Automated Cell Counter, cat. nos. C10281 and C10228)
96‐well microscopy plate (PhenoPlate‐96, TC‐treated, Revvity, cat. no. 6055302)
Plate incubator (e.g., ThermoScientific Series 8000DH) at 37°C, 5% CO_2_, and 85% humidity
Microscope (Operetta CLS high‐content imaging system with Harmony 5.1, Revvity)

#### Day 0: Seed cells

1 Remove spent medium from a 10‐cm plate of Vero cells and rinse plate gently with PBS.
  In our experience, decanting solutions by gently flipping the plate upside‐down results in less cell detachment and loss than using a vacuum aspirator.
2 Incubate at RT for ∼10‐15 min with 1 ml TrypLE until cells are dissociated.
3 Add 5 ml fresh VP‐SFM and transfer to 50‐ml centrifuge tubes.
4 Centrifuge 4 min at 500 × *g* and discard supernatant.
5 Resuspend pellet in fresh VP‐SFM and count total and live cells in an automated cell counter using trypan blue stain.
6 Dilute cells to 25,000 cells/ml with VP‐SFM.
7 Dispense 100 µl into a 96‐well TC‐treated microscopy plate (2500 cells/well).
  We use lower seeding densities as lower confluence allows better visualization of cellular morphology.
  Include single‐color control wells to identify fluorophore crosstalk.
8 Culture overnight in a 37°C incubator.

#### Day 1: Stain cells and acquire data

9 *Optional*: Add heparinase III directly to wells and return to incubator for 1 hr.
  The amount of heparinase needed depends on the source and enzyme activity and thus should be determined empirically.
10 Decant medium and wash wells once with 250 µl fresh VP‐SFM and once with DPBS (with calcium and magnesium).
11 Fix cells with 50 µl cold 4% PFA for 15 min at RT.
12 Decant PFA and wash twice with 250 µl PBS (without calcium and magnesium).
13 Block nonspecific staining by adding 250 µl staining buffer to the cells and incubating for 90 min.
  A blocking step is required to prevent binding of the proteins (antibodies) to the plate.
  The plate can be stored overnight at 4°C as a stopping point.
14 Decant buffer and add primary antibody solutions containing 2 µg anti‐HS scFv in 50 µl staining buffer.
15 Incubate for 30 min at RT.
16 Decant solution and wash twice with 250 µl staining buffer.
  In all subsequent steps, samples must be protected from light to prevent photobleaching.
17 Add 50 µl secondary antibody solution and incubate for 30 min at RT.
18 Decant solution and wash twice with 250 µl staining buffer.
19 Add 50 µl of 10 µg/ml Hoechst 33258 and incubate at RT for 5 min.
  Hoechst 33258 is cell‐permeable and can stain nuclei of unpermeabilized cells.
20 Decant solution, add 100 µl PBS, and proceed to image acquisition.
  Plates can be stored at 4°C before image acquisition. We have stored stained cells for up to 24 hr before imaging.

## Alternate Protocol 3 *IN VITRO* HS DETECTION BY IMMUNOFLUORESCENCE IN SUSPENSION CELLS PRIOR TO FIXATION

**Figure 9:**
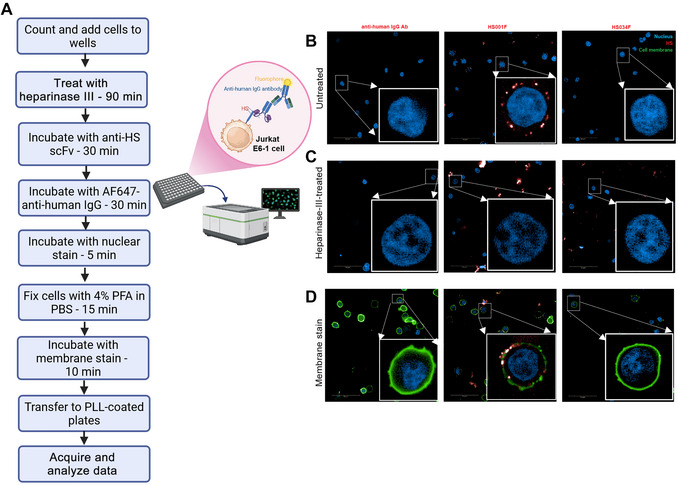
Use of anti‐HS scFvs as staining reagents for immunofluorescence staining of suspension cells. (**A**) Workflow. (**B‐D**) Representative 63× confocal images acquired with Operetta CLS 4 LED system. For each field, an image stack of 15 layers (0.5 µm separation between layers) was acquired with 63× water objective. Scale bars are 50 µm. Nuclei (blue) detected with Hoechst 33258 channel (ex. 355‐385 nm; em. 430‐500 nm). scFv (red) detected with AlexaFluor 647 channel (ex.: 615‐645 nm; em. 655‐760 nm). Cell membranes (green) detected with DiO channel (ex. 530‐560 nm; em. 570‐650 nm). All images were collected with the same acquisition settings and are shown with the same display settings. (**B‐D**) Left to right: secondary antibody control, HS001F, and HS034F (negative control). Projection view of 5 layers of (B) anti‐HS scFv staining, (C) anti‐HS scFv staining after heparinase III treatment, and (D) anti‐HS scFv staining with membrane staining, showing co‐localization of HS001 with membrane stain on the cell surface. HS034 is a negative control scFv and has no known epitope.

This protocol describes staining of live suspension cells followed by fixation. Cells are harvested directly from culture by centrifugation, then treated with heparinase III (to show that the scFvs specifically recognizes HS), washed, and stained for HS (Fig. 9A). Cells are then stained with Hoechst 33258 nuclear stain, fixed, and stained with DiO membrane stain before image acquisition (Fig. 9B‐D).

### Materials

Jurkat E6‐1 cells (ATCC, TIB‐152) maintained in 125‐ml flat‐bottomed flasks per ATCC recommendations, cultured in IMDM with GlutaMAX (Gibco, cat. no. 31980097) and 10% FBS (Sigma, cat. no. 12306C)
0.4% (w/v) trypan blue (Invitrogen, cat. no. T10282)
Heparinase III (lab‐made, New England Biolabs, or Sigma)
Primary antibodies: 2 µg anti‐HS scFv‐F forms in 50 µl staining buffer
Secondary antibody: AlexaFluor‐647‐conjugated anti–human IgG (Invitrogen) diluted 1:250 in staining buffer
10 µg/ml Hoechst 33258: 10 mg/ml stock (Molecular Probes, cat. no. R11496) diluted 1:1000 in PBS
1× PBS, pH 7.4, without calcium or magnesium (Gibco, cat. no. 70011‐04)
4% (w/v) paraformaldehyde (PFA) in 1× PBS (ChemCruz, cat. no. sc‐281692) at 4°C
Vybrant DiO Cell‐Labeling Solution (Invitrogen, cat. no. V22886) diluted 1:200 in PBS

96‐well conical‐bottom, non‐treated polystyrene plates (Nunc, cat. no. 249570) and lids (Corning, cat. no. 3098)
Clear seal (Sigma‐Aldrich, cat. no. Z369659)
Shaking plate incubator (e.g., Climo‐Shaker ISF‐4X) at 37°C, 300 rpm, 8% CO_2_ and 85% humidity
Plate shaker (e.g., Ika MTS 2/4)
Vacuum aspirator (Fisher Scientific, cat. no. NC1472019)
PLL‐coated microscopy plate (see recipe)

#### Prepare cells

1 Count Jurkat E6‐1 cells with an automated cell counter using trypan blue stain and dilute to 5 × 10^5^ cells/ml in fresh medium.
2 Dispense 100 µl into a 96‐well conical‐bottom microplate (50,000 cells/well).
  Include single‐color control wells to identify fluorophore crosstalk.
  All plate shaking steps should be carried out with some form of plate seal (e.g., clear seal) to prevent sample cross‐contamination.
3 *Optional*: Add heparinase III directly to the wells and return to the incubator for 1 hr.
  The amount of heparinase needed depends on the source and enzyme activity and thus should be determined empirically.
  All subsequent steps are performed with cold reagents and at 4°C.
4 Centrifuge 3 min at 500 × *g* and decant the supernatant.

#### Label HS

5 Wash by resuspending cells in 150 µl staining buffer and shaking for 3 min on a plate shaker at 600 rpm.
6 Centrifuge 3 min at 500 × *g* and discard the supernatant.
7 Add primary antibody solutions containing 2 µg anti‐HS‐scFv in 50 µl staining buffer.
8 Resuspend cells for 3 min on a plate shaker at 600 rpm, then reduce speed to 300 rpm and incubate for 30 min.
9 Centrifuge 3 min at 500 × *g* and discard the supernatant.
10 Wash twice as in steps 5‐6.
  In all subsequent steps, samples must be protected from light to prevent photobleaching.
11 Add 50 µl secondary antibody solution.
12 Resuspend cells for 3 min on a plate shaker at 600 rpm, then reduce speed to 300 rpm and incubate for 30 min.
13 Centrifuge 30 min at 500 × *g* and discard the supernatant.
14 Wash twice as in steps 5‐6.
  Unless specified otherwise, the subsequent steps are carried out at RT with RT reagents.

#### Label nuclei and membranes

15 Add 50 µl of 10 µg/ml Hoechst 33258 and incubate for 5 min on a plate shaker.
  Hoechst 33258 is cell‐permeable and can stain nuclei of non‐permeabilized cells.
16 Centrifuge 3 min at 500 × *g* and discard the supernatant.
17 Wash by resuspending cells in 150 µl PBS and shaking on a plate shaker for 3 min.
18 Centrifuge 3 min at 500 × *g* and discard the supernatant.
19 Fix cells with 50 µl cold 4% PFA for 15 min at RT on a plate shaker at 300 rpm.
20 Centrifuge 5 min at 500 × *g* and discard the supernatant.
  To avoid cell loss post fixation, cells should be centrifuged for 5 min instead of 3 min and supernatants should be aspirated gently with a vacuum aspirator.
21 Wash by resuspending cells in 150 µl PBS and shaking on a plate shaker at 600 rpm for 3 min.
22 Centrifuge 5 min at 500 × *g* and discard the supernatant.
23 Repeat wash once more.
24 *Optional*: Stain membranes by adding 50 µl of 1:200 DiO in PBS and incubating for 10 min at RT on a plate shaker at 600 rpm. Wash twice as in steps 21‐22.
25 Resuspend cells in 100 µl PBS for 3 min on a plate shaker at 600 rpm.
26 Transfer cells to a PLL‐coated immunofluorescence‐compatible 96‐well plate.
  A PLL or similar coating on the imaging plate aids in accumulating cells at the bottom of the plate for better imaging.
27 Centrifuge 3 min at 100 × *g* to allow cells to settle at the bottom of the plate.
28 Proceed to image acquisition.
  Plates can be stored at 4°C before image acquisition. We have stored stained cells for up to 24 hr before imaging.

## Reagents and Solutions

### 2×YT medium

*For base medium*:
900 ml H_2_O
16 g tryptone (Fisher Scientific, cat. no. BP9726‐5)
10 g yeast extract (Acros Organics, cat. no. AC451120050)
5 g NaCl (Sigma‐Aldrich, cat. no. S988)
Bring volume to 1 L
Sterilize by autoclaving at 121°C for 15 min
Store up to 4‐6 months at room temperature
*For supplemented medium*:
  Prepare fresh before each use. When glucose is called for, add 10 ml of 20% (w/v) glucose stock (see recipe) to 90 ml sterile base medium for a final 2% (w/v) glucose. For antibiotics, add 34 µg/ml chloramphenicol and/or 50 µg/ml kanamycin (see recipes for stocks) after autoclaving and allowing the medium to cool.

### Antifoam solution, 10% (v/v)

Mix 10 ml Antifoam 204 (Sigma, cat. no. A6426) with 90 ml H_2_O. Sterilize by autoclaving at 121°C for 15 min. Store at room temperature.

### Chloramphenicol stock solution, 34 mg/ml

Dissolve 340 mg chloramphenicol (GoldBio, cat. no. C‐105‐100) in 10 ml of 100% ethanol (Decon, cat. no. 22‐032601). Store aliquots up to 12 months at −20°C.

### ELISA blocking buffer

1× PBS, pH 7.4, without calcium or magnesium (Gibco, cat. no. 70011‐04)
0.005% (v/v) Tween 20 (Sigma, cat. no. P1379)
3% (w/v) bovine serum albumin (BSA, fraction V, RPI, cat. no. A30075‐100.0)
Prepare fresh before each use

### ELISA wash buffer

1× PBS, pH 7.4, without calcium or magnesium (Gibco, cat. no. 70011‐04)
0.1% (v/v) Tween 20 (Sigma, cat. no. P1379)
Store up to 6 months at room temperature

### Ethanol for SEC, 20% (v/v)

Mix 200 ml of 100% ethanol (Decon, cat. no. 22‐023601) and 800 ml water. Filter using a 1000‐ml vacuum filtration system (GenClone, cat. no. 25‐229) and then degas for ~20 min under vacuum. Prepare fresh before each use.

### FACS buffer

1× PBS, pH 7.4, without calcium or magnesium (Gibco, cat. no. 70011‐04)
2% (v/v) FBS
2 mM EDTA
Sterilize using a 0.22‐µm, 500‐ml vacuum filtration system (GenClone, cat. nos. 25‐227)
Store up to 2 weeks at 4°C
  Alternatively, 0.5% (w/v) BSA can be used instead of FBS.

### Glucose stock solution, 20% (w/v)

Dissolve 50 g d‐(+)‐glucose monohydrate (MilliporeSigma, cat. no. 4074) in 100 ml H_2_O. Sterilize using a 0.22‐µm filter. Store up to 12 months at 4°C.

### H_2_O for SEC

Filter using a 1000‐ml vacuum filtration system (GenClone, cat. no. 25‐229) and then degas for ~20 min under vacuum. Prepare fresh before each use.

### His60 elution buffer

25 mM MES (Thermo Fisher, cat. no. H56472.36)
150 mM NaCl (Sigma, cat. no. S9888)
10% (v/v) glycerol (Sigma, cat. no. G7757)
100 mM arginine (Thermo Fisher, cat. no. A14730.0E)
500 mM imidazole (Thermo Fisher, cat. no. A10221.0E)
Adjust pH to 6.5
Sterilize using a 500‐ml vacuum filtration system (GenClone, cat. no. 25‐227)
Store up to 3 months in the dark at room temperature

### His60 slurry, 50% (v/v)

Prepare His60 Ni Superflow resin (Clontech, cat. no. 635662) by removing the storage solution, washing in H_2_O, and resuspending in His60 wash buffer (see recipe) to give a 50% (v/v) slurry. Prepare fresh before each use.

### His60 wash buffer

25 mM MES (Thermo Fisher, cat. no. H56472.36)
150 mM NaCl (Sigma, cat. no. S9888)
10% (v/v) glycerol (Sigma, cat. no. G7757)
50 mM arginine (Thermo Fisher, cat. no. A14730.0E)
5 mM imidazole (Thermo Fisher, cat. no. A10221.0E)
Adjust pH to 6.5
Sterilize using a 500‐ml vacuum filtration system (GenClone, cat. no. 25‐227)
Store up to 3 months in the dark at room temperature

### Immunofluorescence staining buffer

1× PBS, pH 7.4, without calcium or magnesium (Gibco, cat. no. 70011‐04)
1% (w/v) bovine serum albumin (BSA, fraction V, RPI, cat. no. A30075‐100.0)
Sterilize using a 1000‐ml vacuum filtration system (GenClone, cat. no. 25‐229)
Store up to 2 weeks at 4°C

### IPTG stock solution, 1 M

Dissolve 2.38 g isopropyl β‐d‐1‐thiogalactopyranoside (IPTG; GoldBio, cat. no. I2481C100) in 10 ml H_2_O. Sterilize using a 0.22‐µm filter. Store aliquot up to 12 months at −20°C.

### Kanamycin stock solution, 50 mg/ml

Dissolve 500 mg kanamycin monosulfate (GoldBio, cat. no. K‐120‐100) in 10 ml H_2_O. Sterilize using a 0.22‐µm filter. Store in aliquots up to 6 months at −20°C.

### LB agar plates

Dissolve 2.5 g LB medium (Teknova, cat. no. L9145) and 1.5 g agar (Sigma, cat. no. A1296) in 80 ml H_2_O. Bring volume to 100 ml. Sterilize by autoclaving at 121°C for 15 min. After cooling, supplement 90 ml sterile LB agar with 10 ml of 20% (w/v) glucose (see recipe), 34 µg/ml chloramphenicol and 50 µg/ml kanamycin. Pour ∼4 ml/well into 8‐well culture plates (Thermo Fisher Scientific, cat. no. 267062). Allow to harden. Store up to 2 weeks at 4°C.

### NaOH for SEC, 0.5 M

Dissolve 20 g NaOH (Sigma, cat. no. 221465) in 1 L H_2_O. Filter using a 1000‐ml vacuum filtration system (GenClone, cat. no. 25‐229) and then degas for ~20 min under vacuum. Prepare fresh before each use.

### PBST (for ELISA)

1× PBS, pH 7.4, without calcium or magnesium (Gibco, cat. no. 70011‐04)
0.005% (v/v) Tween 20 (Sigma, cat. no. P1379)
Store up to 6 months at room temperature

### PLL‐coated microscopy plate

Dilute 0.01% poly‐l‐lysine hydrobromide (PLL, Sigma, cat. no. P4832) to 0.005% with H_2_O. Add 50 µl per well to a 96‐well microscopy plate (PhenoPlate‐96, TC‐treated, Revvity, cat. no. 6055302) and incubate for 1 hr at 37°C. Rinse twice with 300 µl PBS (Gibco, cat. no. 70011‐04). Store up to 2 weeks at 4°C.

### scFv‐F SEC running buffer

1× PBS, pH 7.4, without calcium or magnesium (Gibco, cat. no. 70011‐04)
2% (v/v) glycerol (Sigma, cat. no. G7757)
0.1 M arginine (Thermo Fisher, cat. no. A14730.0E)

Prepare fresh before each use. Sterilize using a 0.22‐µm, 1000‐ml vacuum filtration system (GenClone, cat. no. 25‐229). Degas for ~20 min under vacuum.

### scFv‐H resuspension buffer

200 mM Tris, pH 8.0 (Sigma, cat. no. T1503)
500 mM sucrose (Sigma, cat. no. S0389)
1 mM EDTA (Sigma, cat. no. E6511)
Sterilize using a 1000‐ml vacuum filtration system (GenClone, cat. no. 25‐229)
Store up to 3 months at 4°C
Before each use, add 1 tablet of complete EDTA‐free Protease Inhibitor Cocktail (Sigma, cat. no. 11873580001) per 200 ml buffer

### scFv‐H SEC running buffer

20 mM HEPES, pH 7.4 (VWRB30487)
150 mM NaCl (Sigma, cat. no. S9888)
10% (v/v) glycerol (Sigma, cat. no. G7757)
Sterilize using a 0.22‐µm filter
Degas for ~20 min under vacuum
Store up to 4 weeks at 4°C

### SDS‐PAGE running buffer

*For 20× running buffer*:
195.2 g MES hydrate (Thermo Fisher, cat. no. H56472.36)
121.14 g Trizma (Sigma, cat. no. T1503)
20 g SDS (Sigma, cat. no. L6026)
6 g EDTA (Sigma, cat. no. E6511)
Adjust volume to 1 L
Dilute to 1× before use
  The final 1× buffer contains 50 mM MES, 50 mM Tris, 0.1% (w/v) SDS, 1 mM EDTA.

### SDS‐PAGE staining solution

Dissolve 80 mg Coomassie brilliant blue G‐250 in 1 L H_2_O and stir for 4 hr. Add 3 ml conc. HCl and stir for 10 min. Store at room temperature.

The original reference (Lawrence & Besir, 2009) states that this can be used for months. We have used it for ~4 months.

## COMMENTARY

### Understanding Results

**Figure 10:**
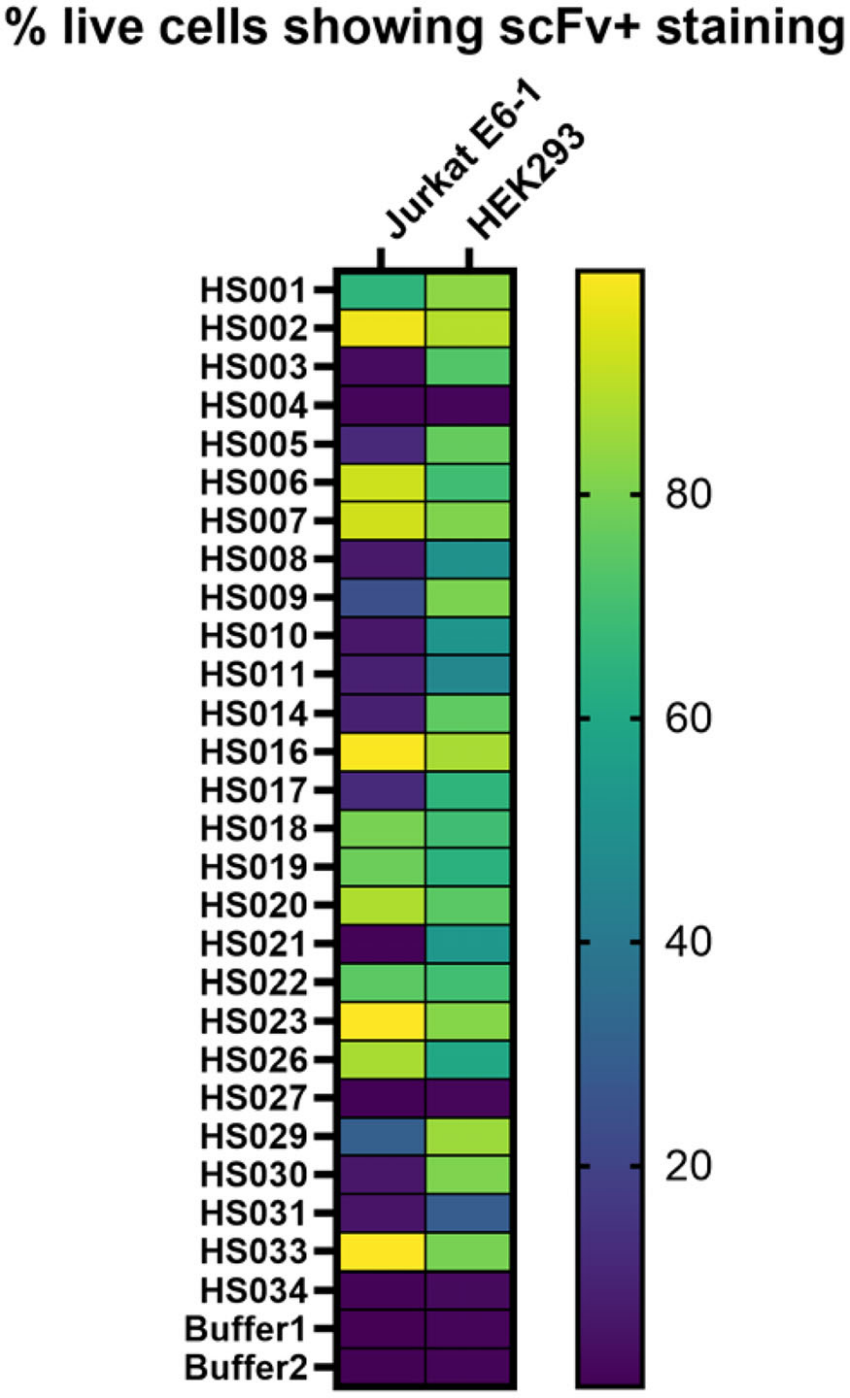
Data for qualitative validation of the anti‐HS scFv‐H panel. Representative data for % live cells showing scFv staining observed for HEK293 and Jurkat E6‐1 cells, obtained following indirect FACS staining as in Basic Protocol 5).

*Benchmarking the anti‐HS scFv panel*. Figure 10 shows representative data as heatmaps for % live cells showing scFv staining obtained using the FACS staining protocol detailed in Basic Protocol 5 for two cell lines: HEK293 cells (ATCC, CRL‐1573, maintained in DMEM supplemented with l‐glutamine, glucose, sodium pyruvate, and 10% FBS) and Jurkat E6‐1 cells (ATCC, TIB‐152, maintained in IMDM with GlutaMAX and 10% FBS). These data are intended to serve as a benchmark for qualitative comparison that researchers can use to ensure that their results align with those from our lab.

*Limitations*. While the protocols described here were optimized for production of the existing anti‐HS scFv panel, it is important to bear in mind that these scFvs are still unique proteins, and some scFvs might require additional optimizations. Additionally, not all antibodies can be used for all techniques. For example, there is a report of antibodies working well to detect epitopes using FACS or immunofluorescence, but not using immunoblotting (Rhodes & Trimmer, 2006).

### Author Contributions

**Kheerthana Duraivelan**: Conceptualization; methodology; validation; investigation; writing—original draft; writing—review and editing. **Sriram Sundaravel**: Conceptualization; methodology. **Esther N. Njoroge**: Methodology. **Robert A. Townley**: Conceptualization; methodology. **Ulrich G. Steidl**: Conceptualization; funding acquisition. **Hannes E. Bülow**: Conceptualization; funding acquisition; writing—review and editing. **Steven C. Almo**: Conceptualization; supervision; funding acquisition; writing—review and editing. **Scott J. Garforth**: Conceptualization; supervision; writing—review and editing.

### Conflict of Interest

Hannes E. Bülow, Ulrich G. Steidl, Steven C. Almo, and Robert A. Townley disclose the following patent application: *Antibody‐based method to identify, purify, and manipulate cell types and processes* (U.S. patent publication no.: US 2022/0227886 A1).

## Data Availability

All data used to prepare this manuscript are contained herein.
